# Vitiligo Signature‐Based Drug Screening Identifies Fulvestrant as a Novel Immunotherapy Combination Strategy

**DOI:** 10.1002/advs.202503979

**Published:** 2025-09-20

**Authors:** Jie Zhu, Liting Huang, Suzhen Bi, WeiKaixin Kong, Wanting Feng, Yingjia Li, Zhengwei Xie, Peipei Shan, Sujie Zhu

**Affiliations:** ^1^ Institute of Translational Medicine The Affiliated Hospital of Qingdao University College of Medicine Qingdao University Qingdao 266071 P. R. China; ^2^ Institute for Molecular Medicine Finland (FIMM) HiLIFE University of Helsinki Helsinki 00100 Finland; ^3^ Peking University International Cancer Institute Peking University‑Yunnan Baiyao International Medical Research Center State Key Laboratory of Natural and Biomimetic Drugs Department of Molecular and Cellular Pharmacology School of Pharmaceutical Sciences Peking University Health Science Center Beijing 100191 P. R. China

**Keywords:** Fulvestrant, immunotherapy, vitiligo

## Abstract

Immunotherapy has revolutionized cancer treatment; however, only 10–30% of patients experience durable survival benefits, while most malignancies remain resistant. In melanoma, vitiligo‐like depigmentation is a frequent and generally mild immune‐related adverse event, whose presence correlates positively with enhanced antitumor immune responses and prolonged patient survival. By performing comparative analyses between vitiligo and melanoma, we established a biomarker panel—designated the vitiligo signature (VGS)—that differentiates “cold” from “hot” tumors with high accuracy. Leveraging a deep learning–based efficacy prediction system (DLEPS), we identified and validated Fulvestrant as a candidate capable of enhancing anti–programmed cell death ligand 1 (PD L1) therapy in preclinical models. Single cell RNA sequencing revealed that Fulvestrant expanded cytotoxic T cell populations, while immunofluorescence and flow cytometry confirmed markedly increased CD8⁺ T cell infiltration into tumor tissue. Mechanistic investigations demonstrated that Fulvestrant activates the C─C motif chemokine 5 (CCL5), major histocompatibility complex class I (MHC I), and type II interferon (IFN II) signaling pathways, thereby potentiating antitumor immunity. Collectively, our study introduces a precision approach for patient stratification in immunotherapy and highlights Fulvestrant as a promising component of immunotherapy based combination strategies warranting clinical evaluation.

## Graphical abstract

A vitiligo‐derived gene signature predicts response to immune checkpoint blockade across multiple cancer cohorts, providing a framework for patient stratification. Building on this, Fulvestrant—a clinically approved estrogen receptor degrader—further enhances antitumor immunity by reprogramming tumor‐associated macrophages and promoting CD8⁺ T cell infiltration, suggesting a potential combination strategy to improve immunotherapy efficacy.

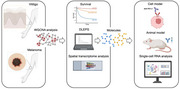

## 1 Introduction

Immunotherapy has revolutionized cancer treatment, particularly through immune checkpoint blockade (ICB), which targets regulatory pathways such as programmed cell death protein 1 (PD1)/ programmed cell death ligand 1 (PD‐L1) and cytotoxic T‐lymphocyte protein 4 (CTLA4).^[^ ^1^ ^]^ These therapies have demonstrated remarkable efficacy, achieving durable clinical responses in certain cancers, including melanoma and non‐small‐cell lung cancer.^[^ ^1^, ^2^, ^3^ ^]^ However, despite the transformative potential of ICB, the majority of patients with advanced cancers derive limited or no benefit from these therapies due to the complexity and heterogeneity of the tumor immune microenvironment.^[^ ^4^, ^5^ ^]^ This variability presents a substantial obstacle, emphasizing two critical challenges: identifying biomarkers to predict patient response to ICB, and developing therapeutic strategies to enhance ICB effectiveness in non‐responding patients.

Current efforts to address these challenges include exploring predictive biomarkers, such as PD‐L1 expression^[^ ^6^ ^]^ and tumor mutational burden (TMB),^[^ ^7^ ^]^ which have shown some correlation with treatment efficacy. However, these biomarkers lack universality and precision across different cancer types and patient populations. Consequently, there is an urgent need for more robust and widely applicable biomarkers. Emerging evidence suggests that combining ICB with other agents that augment T cell activation may enhance treatment outcomes.^[^ ^8^ ^]^ For instance, the androgen receptor (AR) inhibitor NACT02312557 has shown potential in increasing patient response rates when administered alongside PD‐1 blockade,^[^ ^9^ ^]^ underscoring the promise of combination therapies to potentiate T cell responses and broaden ICB applicability. Despite this promise, the identification of effective biomarker‐driven strategies to guide such combination treatments remains an unmet need.

Interestingly, immune‐related adverse events (irAEs) observed during immunotherapy have provided insights into the potential of immune activation as a predictive factor for therapeutic outcomes. These irAEs, arising from off‐target immune activation, which called “beneficial autoimmunity” have shown association with favorable prognosis and robust anti‐tumor responses in certain cancer types.^[^ ^10^, ^11^, ^12^ ^]^ Specifically, vitiligo, a mild form of irAE characterized by skin depigmentation, has been observed in melanoma patients undergoing ICB and is associated with improved survival.^[^ ^13^, ^14^, ^15^, ^16^ ^]^ The occurrence of vitiligo in patients receiving anti‐CTLA‐4 and anti‐PD‐1 therapies ranges from 2–11% and 8–25.7%, respectively.^[^ ^17^, ^18^, ^19^ ^]^ Importantly, the immunological mechanisms underlying vitiligo closely resemble those observed in inflamed, immunotherapy‐responsive tumors. Both conditions are marked by enhanced CD8⁺ T cell infiltration,^[^ ^20^ ^]^ interferon‐gamma (IFN‐γ) pathway activation,^[^ ^21^ ^]^ and chemokine‐driven recruitment of effector T cells, including C─X─C motif chemokine 9 (CXCL9), C─X─C motif chemokine 10 (CXCL10), and CCL5.^[^ ^22^, ^23^, ^24^ ^]^ Moreover, both vitiligo lesions and T cell–inflamed tumors exhibit elevated expression of antigen presentation machinery, MHC class I molecules, and interferon‐stimulated gene signatures. These converging features suggest that vitiligo represents a naturally occurring model of productive, antigen‐specific T cell immunity. From a systems‐level perspective, the transcriptional landscape of vitiligo mirrors that of the tumor immune microenvironment in patients who benefit from ICB. Thus, studying the gene expression programs active in vitiligo may uncover clinically relevant biomarkers reflective of favorable immune engagement in cancer. These features align with the conditions favorable for an effective anti‐tumor immune response, potentially explaining the observed association between vitiligo and improved clinical outcomes in melanoma.

Although vitiligo can act as an effective marker for immune therapy, the response typically emerges only after a considerable period of immune therapy and is not suitable as an effective marker for screening patients for immune therapy before treatment. The difference in gene expression serves as the main cause of the heterogeneity of the tumor immune microenvironment. Thus, we hypothesized that genes associated with vitiligo may indicate heightened immune activation and could serve as predictive biomarkers of favorable immunotherapy outcomes. Additionally, we proposed that pharmacological induction of a “vitiligo‐like” immune signature in tumors could improve responsiveness to ICB. To test these hypotheses, we identified a set of genes, termed the Vitiligo Signature (VGS), through integrated analysis of data from vitiligo, melanoma, and anti‐PD‐1 immunotherapy cohorts. Our results indicate that high VGS expression is associated with features of an immune‐responsive tumor microenvironment, including increased tumor‐infiltrating lymphocytes (TILs), higher expression of immune checkpoints, elevated TMB, and a T cell‐inflamed gene expression profile (GEP).^[^ ^25^ ^]^ Spatial transcriptomics further demonstrated that patients with elevated VGS expression had improved responses to immunotherapy, suggesting that VGS may serve as a predictive biomarker of enhanced survival outcomes in melanoma.

To translate these findings into therapeutic applications, we utilized the deep learning–based efficacy prediction system (DLEPS) platform, a deep‐learning‐based drug screening tool,^[^ ^26^ ^]^ to identify compounds capable of inducing VGS‐associated immune activation. Among the screened agents, Fulvestrant, a selective estrogen receptor degrader (SERD),^[^ ^27^ ^]^ emerged as a promising candidate for combination therapy. Preclinical models demonstrated that Fulvestrant significantly enhanced the efficacy of anti‐PD‐L1 therapy in multiple cancers, including melanoma, breast cancer, and colon cancer. Mechanistic studies revealed that Fulvestrant treatment increased CD8+ T cell infiltration and activated antigen‐processing pathways, including CXCL, MHC‐I, and IFN‐II signaling. Immunofluorescence and flow cytometry further confirmed the increased recruitment of cytotoxic CD8+ T cells within tumors, supporting Fulvestrant's potential as an immune‐enhancing agent in combination with ICB.

In conclusion, in this study, we identified the VGS as a potential biomarker with predictive utility for ICB responsiveness from the “beneficial autoimmunity” in a new perspective, which may facilitate patient selection for immunotherapy. While existing biomarkers such as PD‐L1 expression and tumor mutational burden (TMB) offer limited predictive value and often lack consistency across cancer types, the VGS‐based approach captures key features of effective anti‐tumor immunity derived from naturally occurring immune activation, providing a biologically grounded and broadly applicable alternative. Furthermore, the identification of Fulvestrant as an inducer of vitiligo‐like immune activation underscores a novel therapeutic approach for enhancing ICB efficacy, leveraging beneficial autoimmune responses to improve clinical outcomes in cancer patients, and this approach seeks will provide strategies for combination immunotherapy protocols and prospective clinical development. Notably, the proposed VGS‐guided drug repurposing framework represents a biologically informed and tumor‐agnostic approach, uniquely grounded in naturally occurring immune activation. By integrating transcriptional signatures with deep learning–based compound screening, this method enables both precise prediction of ICB responsiveness and the systematic identification of agents capable of reprogramming the tumor immune microenvironment to favor therapeutic response.

## 2 Results

### 2.1 The Integrated Analysis between Vitiligo Progression and the Survival of Melanoma Patients Demonstrated a Valid Vitiligo Gene Signature

To investigate biomarkers in response to immunotherapy, we performed WGCNA analysis (Figure S1A, Supporting Information) and observed that the dark orange and blue modules were significantly correlated with the progression of vitiligo (cor = −0.89, *p*‐value < 0.0001, and cor = 0.78, *p*‐value < 0.0001, respectively) (Figure S1B, Supporting Information). We also identified 1450 differentially expressed genes (DEGs) with |log(foldchange)| >0.5 and *p*‐value < 0.05 between vitiligo patients and healthy controls. An overlap of 262 genes was observed between the two gene lists obtained by the WGCNA method and DEGs, and these genes were characteristic genes associated with vitiligo progression (Figure S1C, Supporting Information).

**Figure 1:**
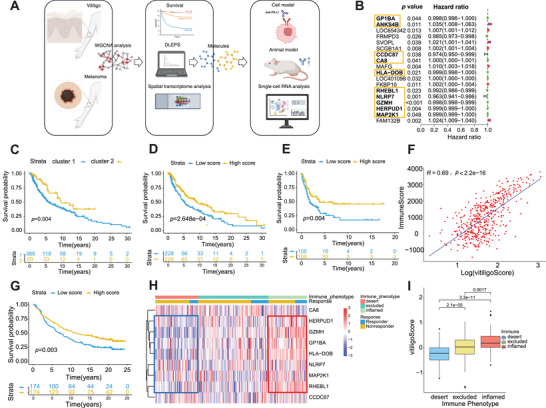
The analysis of the relationship between vitiligo and melanoma. A) The workflow of this study. B) the prognostic value of 18 genes (10 genes marked) with p‐value less than 0.05 among 262 overlapping genes between DEGs and the genes obtained by the WGCNA method, and statistic significance was obtained by the Wald test. C) the survival plot of two clusters in the TCGA cohort, and statistical significance of the survival differ‐ ences between the two groups was assessed with the Log Rank test. D) the survival plot of low VS‐score and high VS‐score (vitiligo score) in the TCGA cohort. Statistical significance of the survival differences between the two groups was assessed with the Log Rank test, Hazard Ratio: 0.60 (95% CI: 0.46–0.79). E) the survival plot of low VS‐score and high VS‐score in the GSE65904 cohort. Statistical significance of the survival differences between the two groups was assessed with the Log Rank test, Hazard Ratio: 0.57 (95% CI: 0.38–0.84). F) the relationship between immuneScore (ESTIMATE method) and VS in the TCGA cohort (Person correlation). G) the survival plot of low VS‐score and high VS‐score in IMvigor210 cohort. Statistical significance of the survival differences between the two groups was assessed with the Log Rank test, Hazard Ratio: 0.68 (95% CI: 0.52–0.88). H) the heatmap of vitiligo signature genes and the immune phenotype in IMvigor210 cohort. I) the plot of the relation‐ ship between the immune phenotype of VS, Wilcoxon test.

In the next step, we identified 18 genes with prognostic value using univariate Cox regression on the 262 vitiligo‐characteristic genes in the melanoma cohort (**Figure** **1**B). We then aimed to identify consistent genes between melanoma and vitiligo. Given that melanoma patients with vitiligo tend to have better survival, we matched genes that were favorable factors in the Cox regression of melanoma to those that were highly expressed in vitiligo (compared to healthy controls). Similarly, genes that were unfavorable factors in Cox regression were matched to those that were lowly expressed in vitiligo. Finally, we obtained ten matched genes (GP1BA, ANKS4B, CCDC87, CA8, HLA‐DOB, RHEBL1, NLRP7, GZMH, HERPUD1, and MAP2K1) as a vitiligo‐gene signature (VGS) (Figure 1B). To further explore the role of VGS in melanoma, we initially divided the GSE65904 cohort into two clusters based on the expression of VGS (Figure S1D, Supporting Information). As anticipated, the survival states of these two clusters differed, with cluster 2 demonstrating a significant survival advantage and exhibiting high expression of GZMH, RHEBL1, MAP2K1, GP1BA, HLA‐DOB, HERPUD1, and NLRP7 (Figure S1E, Supporting Information; Figure S1G), while cluster 1 was characterized by increased expression of CCDC87, ANKS4B, and CA8. Similar results were observed in the TCGA cohort (Figure S1F,H, Supporting Information; Figure 1C). Notably, cluster 2 displayed a significant increase in the numbers of CD8+ T cells, activated CD4+ T cells, and a decrease in the number of macrophages (Figure S2A, Supporting Information, TCGA cohort). Furthermore, Gene Set Enrichment Analysis (GSEA) showed that immunity was more activated in cluster 2 (Figure S2B,C, Supporting Information, TCGA cohort). Based on these findings, we speculate that patients in cluster 2 have a better prognosis due to a more favorable immune microenvironment and a higher immune response.

### 2.2 Molecular Landscape of Immunity Associated with Vitiligo Score (VS)

Considering the individual heterogeneity and complexity of vitiligo patterns, we constructed a scoring system based on the VGS to quantify the vitiligo patterns of individual patients with melanoma, namely the Vitiligo Score (VS) (Figure S3A, Supporting Information). We observed that the VS in cluster 2 was higher than that in cluster 1 (Figure S3B, Supporting Information). To evaluate the prognostic significance of the VS, we performed Kaplan–Meier survival analysis across two independent cohorts: the TCGA melanoma cohort and a GEO validation dataset. Patients were stratified into VS‐high and VS‐low groups using the median VS as the cutoff. In the **TCGA cohort**, the VS‐high group showed significantly improved overall survival compared to the VS‐low group, with a **median survival time of 3379 days versus 1864 days**, respectively. **Univariate Cox proportional hazards analysis** yielded a **hazard ratio (HR) of 0.60** (95% CI: 0.46–0.79, ***p* = 0.0003**), indicating a 40% reduction in the risk of death in the VS‐high group. **Similarly, in the GEO dataset, the VS‐high group exhibited improved survival with a HR of 0.57 (95% CI: 0.38–0.84, *p* = 0.004)** (Figure 1D,E). Moreover, we found that VS was positively correlated with the immune score calculated by the ESTIMATE algorithm (*R* = 0.69, *p* < 0.001, Figure 1F). Considering that patients with a high VS showed better survival and an activated immune status, we suspect its potential role as a biomarker in immunotherapy.

To better understand the clinical significance of the VS in immunotherapy, we investigated its association with several effective biomarkers in response to immunotherapy. These biomarkers included higher expression of PD‐L1/CTLA‐4, the number of tumor‐infiltrating lymphocytes (TILs), a high TMB, a more dominant T cell‐inflamed GEP, and greater mismatch‐repair deficiency.^[^ ^25^ ^]^ Surprisingly, we found that patients with a high VS demonstrated broad benefits, including higher expression of PD‐L1/CTLA‐4 (Figure S3C, Supporting Information, TCGA cohort), upregulated expression of T cell‐inflamed GEP (Figure S3D, Supporting Information, TCGA cohort), a higher evaluated TMB (Figure S3E–G, Supporting Information, TCGA cohort), and a higher number of CD8+ T cells, plasma cells, and activated CD4+ memory cells, but a lower number of macrophages (Figure S3H, Supporting Information, TCGA cohort). These findings indicate that patients with a high VS exhibit stronger anti‐tumor immune responses and better survival after immunotherapy. Based on these results, we next investigated whether VS could serve as an independent predictor for patient survival in the immunotherapy cohort. As expected, in the IMvigor210 cohort, VS‐high patients had a longer median survival of 4161 days, compared to 2577 days in the VS‐low group. The association remained statistically significant, with a HR of 0.68 (95% CI: 0.52–0.88, *p* = 0.003), suggesting that higher VGS expression is associated with better immunotherapy response and prolonged survival (Figure 1G). Furthermore, patients with a high VS were more likely to achieve complete or partial responses, or have an inflamed tumor microenvironment in response to immunotherapy (Figure 1H,I), indicating that VS could predict immune responses to anti‐PD‐1/L1 immunotherapy. Overall, our analysis suggests that VS has the potential to identify the tumor immunophenotype as well as patients who are more likely to benefit from anti‐PD‐1/L1 immunotherapy.

### 2.3 Spatial Transcriptome Dataset Revealed the Responder of Immunotherapy Exhibited Higher Expression of VGS

**Figure 2:**
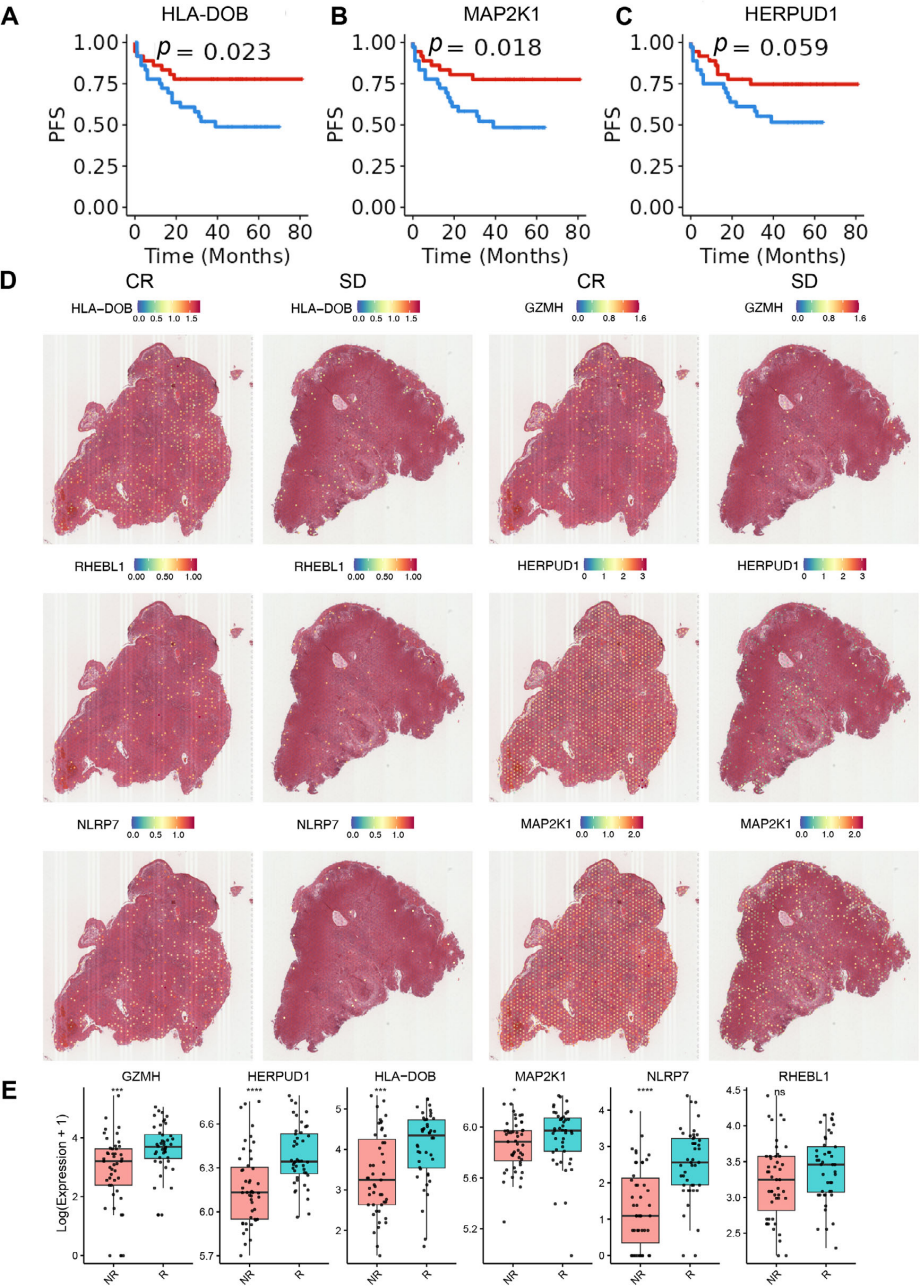
Responder patients in immunotherapy exhibited higher expression of VGS compared with non‐responder patients. A–C) The progression‐free survival of melanoma patients stratified by the expression of HLA‐DOB (A), MAP2K1 (B), and HERPUD1 (C). Log‐rank test. D) the gene expression of HLA‐DOB, GZMH, RHEBL1, HERPUD1, NLRP7, and MAP2K1 in complete response (CR) and stable disease (SD) patients. E) boxplots show the expression of six VGS genes (GZMH, HERPUD1, HLA‐DOB, MAP2K1, NLRP7, and RHEBL1) in immunotherapy responders (R) versus non‐responders (NR) in the Gide et al. dataset. Expression values are shown as Log(Expression + 1). Statistical comparisons were performed using the Wilcoxon rank‐sum test (*p* < 0.05; *p* < 0.01; *p* < 0.001; ns = not significant).

We also explored the prognostic value of the Vitiligo Gene Signature (VGS) in an additional melanoma cohort. Six genes were available in this cohort, and survival analysis revealed that **three of the six genes—MAP2K1, RHEBL1, and HLA‐DOB—were significantly associated with patient survival** (**Figure** **2**A–C; Figure S4A–C, Supporting Information). To assess whether VGS expression is linked to response to immunotherapy, we first analyzed a spatial transcriptomics dataset. All six VGS genes were detected in this dataset, and **their expression levels were significantly higher in patients who achieved complete response (CR) compared to those with stable disease (SD)** (Figure 2D; Figure S4D, Supporting Information), suggesting a potential predictive role for VGS in ICB efficacy.

To further validate these findings, we analyzed the expression of the six VGS genes in **two independent bulk RNA‐seq datasets from ICB‐treated melanoma cohorts**. Consistent with our previous observations, **GZMH, HLA‐DOB, and MAP2K1 were significantly upregulated in responders (R) compared to non‐responders (NR)** in both cohorts^[^ ^28^, ^29^ ^]^ (Figure 2E; Figure S4E, Supporting Information). In one cohort, **HERPUD1 and NLRP7** also showed statistically significant increases in responders, and although RHEBL1 did not reach statistical significance, it displayed a consistent upward trend in the responder group (Figure 2E; Figure S4E, Supporting Information). These results strengthen the association between VGS expression and favorable clinical outcomes in melanoma immunotherapy and support the utility of VGS as a potential predictive biomarker.

After defining the gene expression signature linked with vitiligo progression, named “VGS,” we next went on to identify small molecules that could induce the tumor cells to present with the vitiligo‐like expression signature, with the hypothesis that these candidate drugs may active the antitumor immunity similar to the vitiligo condition to synergize with the ICB treatment. We used these VGS genes (Table S1, Supporting Information) as input for our previously developed DLEPS platform to perform a virtual drug response screening. DLEPS is a deep‐learning based drug efficacy prediction system, which can identify potential agents to treat diverse diseases based on changes of transcriptional profiles as its input.^[^ ^26^ ^]^ The DLEPS score indicates the extent to which specific agents could reverse (negative DLEPS score) or induce (positive DLEPS score) the expression states of the input genes. Therefore, we focused on the agents with the highest positive DLEPS scores to induce the up‐regulation of the vitiligo transcriptomic signature. We chose the Fulvestrant, Motolimod, and Cobicistat for further experiments based on the DLEPS score (Table S2, Supporting Information).

### 2.4 Fulvestrant and Motolimod Identified as the Top‐Synergizer of Immunotherapy to Enhance the Efficacy of ICB Therapy in Solid Tumors

**Figure 3:**
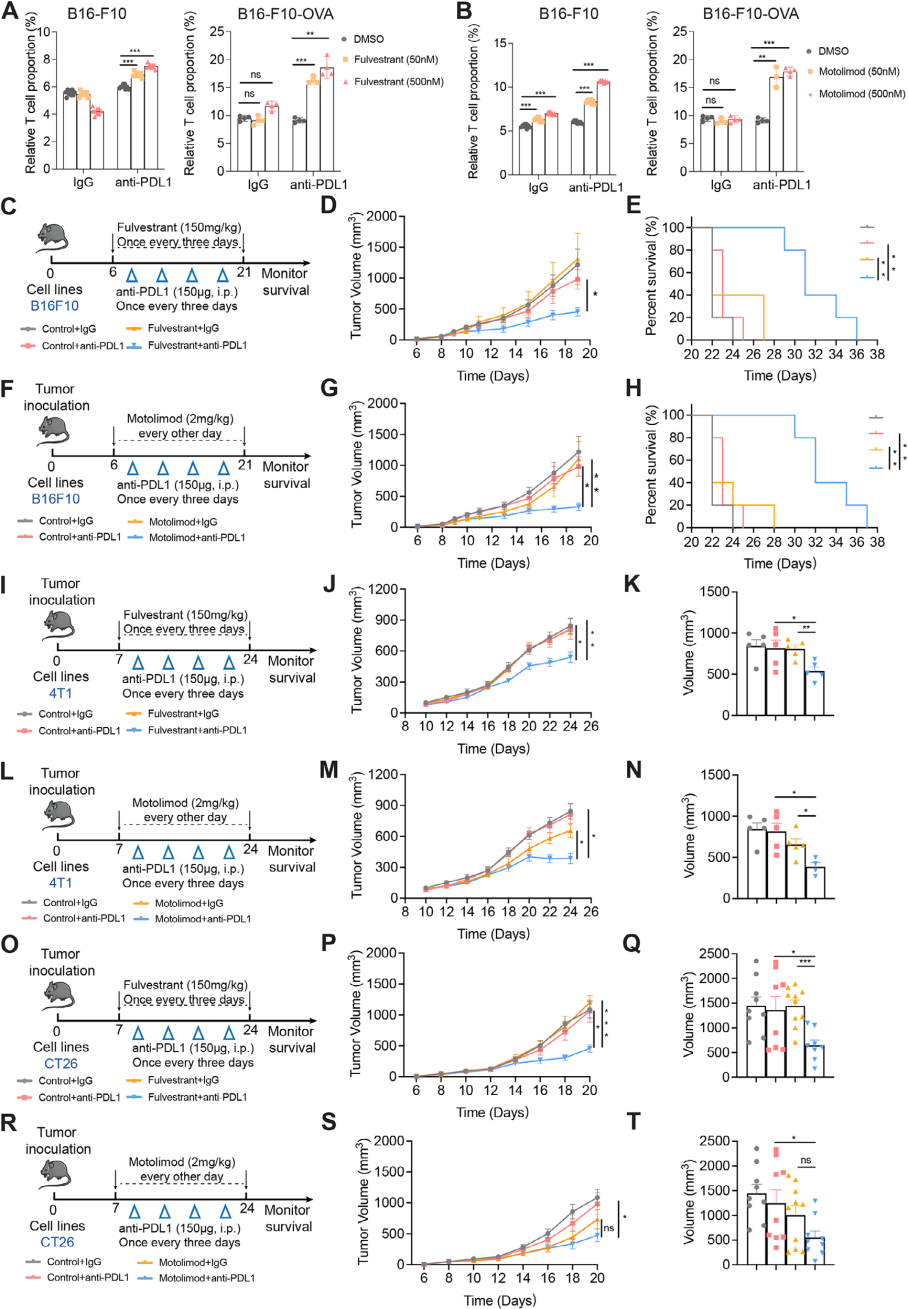
Fulvestrant and Motolimod enhanced the effects of anti‐PDL1 treatment. A) Relative T cell proportion in B16‐F10 cell and B16‐F10‐OVA cell treated with 50 and 500 nM Fulvestrant for 48 h. B) Relative T cell proportion in B16‐F10 cell and B16‐F10‐OVA cell treated with 50 and 500 nM Motolimod for 48 h. C,F,I,L,O,R) Illustration of animal models. B16‐F10 cells were injected into C57BL mice. Animals were administrated when the volumes of tumors were ≈50mm^3^. 4T1 cells and CT26 cells were injected into Balbc mice. Animals were administrated when the volumes of tumors were ≈50 mm^3^. D,G,J,M,P,S) Mean tumor volume (mm^3^) of B16‐F10, 4T1, and CT26 during treatment with Fulvestrant (150 mg kg^−1^) and Motolimod (2 mg kg^−1^) or the combination. n=5 tumors. The growth of B16‐F10 tumors, 4T1 tumors, and CT26 tumors were measured by tumor volume; volume (mm^3^) = [width^2^ (mm^2^) × length (mm)]/2. E,H) Kaplan‐Meier plots demonstrating the association between B16‐F10 tumors and overall survival. K,N,Q,T) Tumor sizes at day 19 (sample‐paired Student's *t*‐test). ^*^ *p* < 0.05, ^**^ *p* < 0.01, ^***^ *p* < 0.001, and ^****^ *p* < 0.0001. Error bars depict SEM.

Since the T cell infiltration contributes to the effects of immunotherapy,^[^ ^30^ ^]^ we first tested the combination effects of the selected four compounds with anti‐PD‐L1 in vitro, and T cell migration experiments demonstrated that Fulvestrant, Motolimod, or Cobicistat with PD‐L1 antibody treatment could significantly enhanced T cell migration compared with PD‐L1 antibody perturbation. Furthermore, we observed that the proportion of T cells migrating increased with the rising drug concentrations (**Figure** **3**A,B; Figure S5A, Supporting Information). Then, we tested the effects of Fulvestrant, Motolimod or Cobicistat in combination with anti‐PDL1 monoclonal antibody in a syngeneic mouse model of melanoma (B16‐F10 with C57BL/6). Remarkably, Fulvestrant and Motolimod were able to enhance the immunotherapy response and extend the lifespan of the treated animals (Figure 3C–H; Figure S5C,D, Supporting Information), while Cobicistat combined with anti‐PDL1 did not reduce tumor growth at treatment period and finally extend the lifespan of treated animals compared with anti‐PDL1 group (Figure S5E–H, Supporting Information). Since Fulvestrant and Motolimod exhibited better efficacy, there two drugs were selected for further studies. And we also observed the expression levels of Gp1ba, Gzmc (human homologous GZMH), and Gzmb genes exhibited a significant increase in the Fulvestrant treatment group when compared to those in the control group, while Motolimod that there was a marked elevation in the expression levels of H2‐Ob (human homologous HLA‐DOB gene) alongside both Gzmc and Gzmb genes (Figure S6A,B, Supporting Information).

To test the combination effects in other solid tumors than melanoma, and to use mouse strains other than C57BL/6, we chose 4T1 breast cancer and CT26 colorectal cancer lines injected into BALB/c mice to build orthogonal tumor mouse models. Similar to the mouse model of melanoma, Fulvestrant or Motolimod enhanced the efficacy of anti‐PDL1 therapy also in breast cancer and colon cancer tumors in vivo (Figure 3F–T). These animal experiments suggest Fulvestrant or Motolimod as a potential co‐inhibitor for enhancing immune checkpoint blockade in solid tumors. Given that Fulvestrant has more extensive clinical applications, we chose Fulvestrant for further studies.

### 2.5 Fulvestrant Increased CD8+ T Lymphocytes into Tumor for Enhancing Anti‐PDL1 Therapy

**Figure 4:**
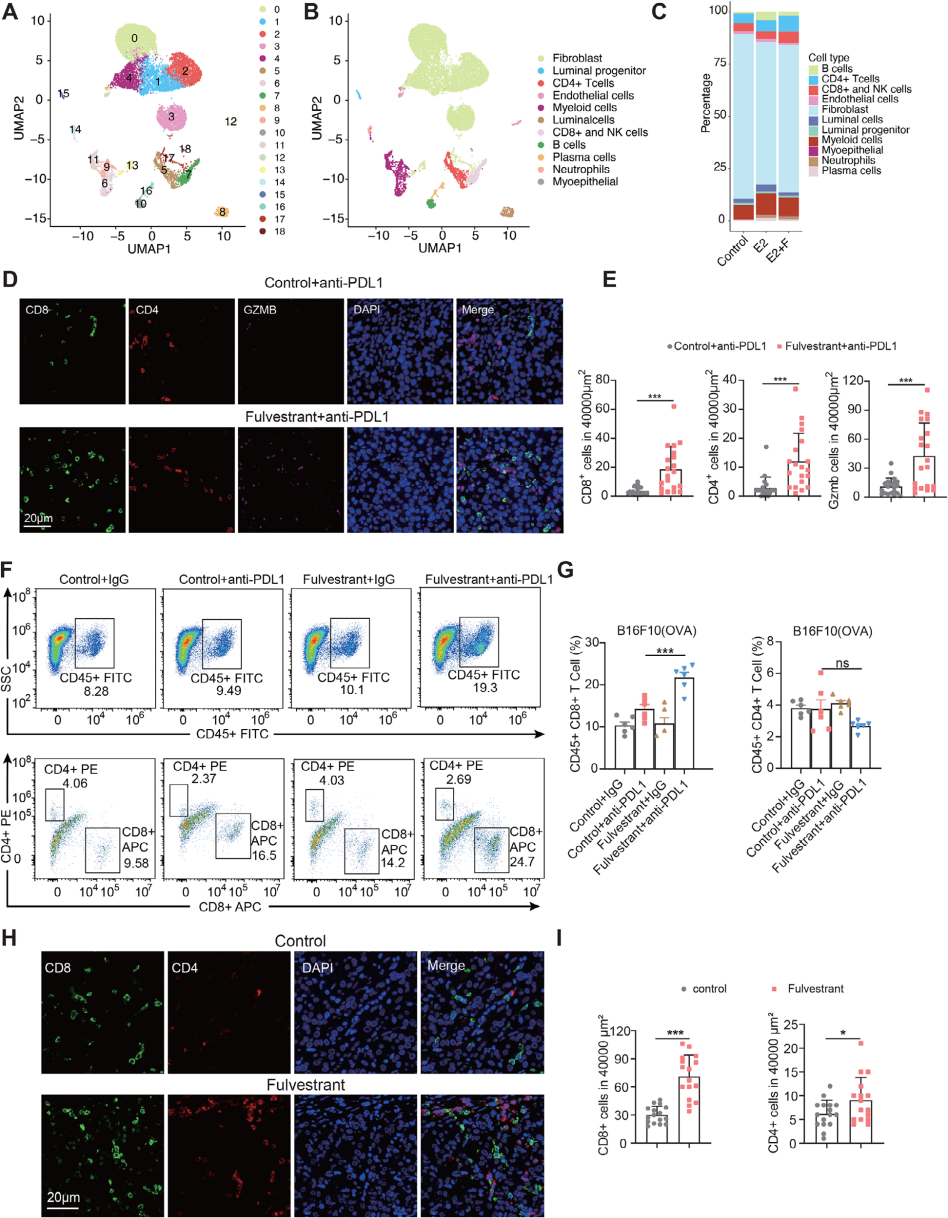
Cytotoxic T cells significantly increased after Fulvestrant perturbation. A) 19 cell clusters were identified by unsupervised clustering. B) 11 cell types were annotated based on established cell markers. C) the differences in proportions of all 11 cell types among control, E2, and E2+F samples. D) Representative images of immunofluorescence (IF) staining showed the distribution of CD8+T cells, CD4+T cells, and GZMB cells in control+anti‐PDL1 and Fulvestrant+anti‐PDL1 treated 4T1 tumors. Scale bar, 20um. E) Statistic analy‐ sis of IF staining results of CD8+ T cells, CD4+ T cells, and GZMB cells in control+anti‐PDL1 and Fulves‐ trant+anti‐PDL1 treated 4T1 tumors. F,G) Representative flow cytometry plots of CD8+ and CD4+ T cells (F) and the percentages of CD8+ and CD4+ T cells within the CD45+ cell population (G) in B16F10 (OVA) xeno‐ graft tumors (*n*=6) after four injections of PD‐L1 antibody in different treatment groups. One‐way ANOVA was used to determine statistical significance. Data are presented as mean ± SD. ^*^ *p* < 0.05; ^**^ *p* < 0.01; ^***^ *p* < 0.001; ^****^ *p* < 0.0001. H) Representative images of IF staining showing the distribution of CD8+ T cells and CD4+ T cells Scale bar, 20um. Control and Fulvestrant treat groups, respectively. I) Representative IF analysis of CD8+ T cells and CD4+ T cells in independent samples.

Previous studies have shown that patients who response to immunotherapy contained more cytotoxic T cells such as CD8+ T cells,^[^ ^31^ ^]^ therefore, we performed single‐cell RNA sequencing analysis, and the cells from the 3 samples including control, estradial (E2) treated, and estradial comined with Fulvestrant (E2+F) treated mouse mammary glands were divided into 19 cell clusters using unsupervised clustering (**Figure** **4**A) according to the transcriptomic profiles. Based on established cell type markers (Table S3, Supporting Information), the 19 cell clusters were assigned to 11 cell types (Figure 4B; Figure S7, Supporting Information). We observed that the proportion of CD8+, CD4+, and NK cells was increased by Fulvestrant compared with E2 treatment (Figure 4C). To confirm our findings from the scRNA‐seq resource, we next performed immunofluorescence staining of B16‐F10 mouse tumor specimens and we observed that Fulvestrant combined with anti‐PDL1 increased the infiltration of CD8+, CD4+, and GZMB cells into the tumors compared to the control group (Figure 4D,E). Furthermore, flow cytometry analysis confirmed that Fulvestrant combined with anti‐PD‐1 significantly enhanced the proportion of CD8+ T cells of B16‐F10 (OVA), 4T1, or CT26 tumors (Figure 4F,G; Figure S8A–D, Supporting Information). These findings suggest that Fulvestrant could promote CD8+ T cell infiltration synergistically with anti‐PD‐1 therapy to improve the immune response against the tumor. Moreover, we collected two breast patients with or without Fulvestrant treatment, we observed that Fulvestrant treatment significantly increased CD4+ and CD8+ T cells infiltration compared with control breast patients (Figure 4H,I).

**Figure 5:**
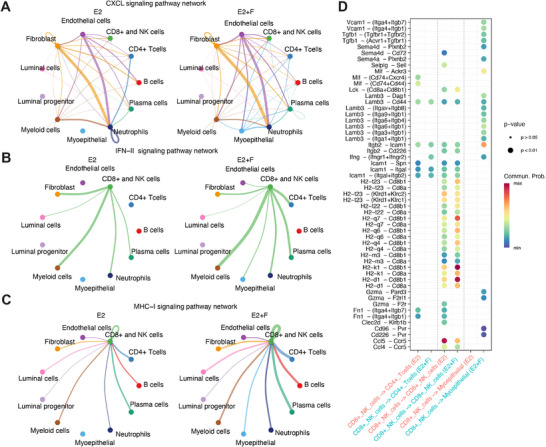
Fulvestrant significantly activated the antigen processing and presentation signaling pathways. A–C) the differences of cell communication strength between the E2 and E2+F group in CXCL, IFN‐II, and MHC‐I signaling pathways. After Fulvestrant perturbation, the cell communication of the above three pathways was increased for the cytotoxic T cells (CD8+ and NK cells) and myoepithelial cells. D) the differences in communication probability between cytotoxic T cells and CD4 T cells or myo‐ epithelial cells.

Having identified different types of cells, we next explored the cell communication between the E2 group and E2+F group, and we especially focused on the CXCL, IFN‐II, and MHC‐I signaling pathway, since these pathways are important for the antigen processing and presentation.^[^ ^32^, ^33^, ^34^ ^]^ We found that Fulvestrant treatment significantly increased the communication between myoepithelial and CD8+ and NK cells (**Figure** **5**A–C; Figure S9A–F, Supporting Information), and previous studies have shown myoepithelial cells have anti‐tumor ability.^[^ ^35^ ^]^ Notably, the genes related to antigen expression (such as H2‐k1, H2‐d1, and H2‐q7) were increased in the E2+F group when compared with the E2 group (Figure 5D). All of these results supported that Fulvestrant could enhance the activities of cytotoxic T cells to enhance the antitumor effects.

### 2.6 Fulvestrant Reprograms M2‐Like Macrophages and Facilitates CD8⁺ T Cell Infiltration

To investigate how fulvestrant enhances cytotoxic T cell infiltration, we first examined whether it induces a stronger immune response through direct cancer cell killing and subsequent tumor antigen (TA) release, and we performed in vitro drug sensitivity assays in 4T1, B16‐F10, and CT26 tumor cell lines. Dose–response survival curves showed no significant reduction in viability following Fulvestrant treatment, even at high concentrations (Figure S10A–F, Supporting Information). Similarly, crystal violet staining and colony formation assays demonstrated no meaningful suppression of tumor cell clonogenic potential (Figure S10G, Supporting Information). In addition, the transwell migration assays revealed that Fulvestrant did not significantly impair the migratory ability of tumor cells (Figure S10H, Supporting Information). Collectively, these results indicate that Fulvestrant does not directly affect tumor cell viability, proliferation, or migration.

**Figure 6:**
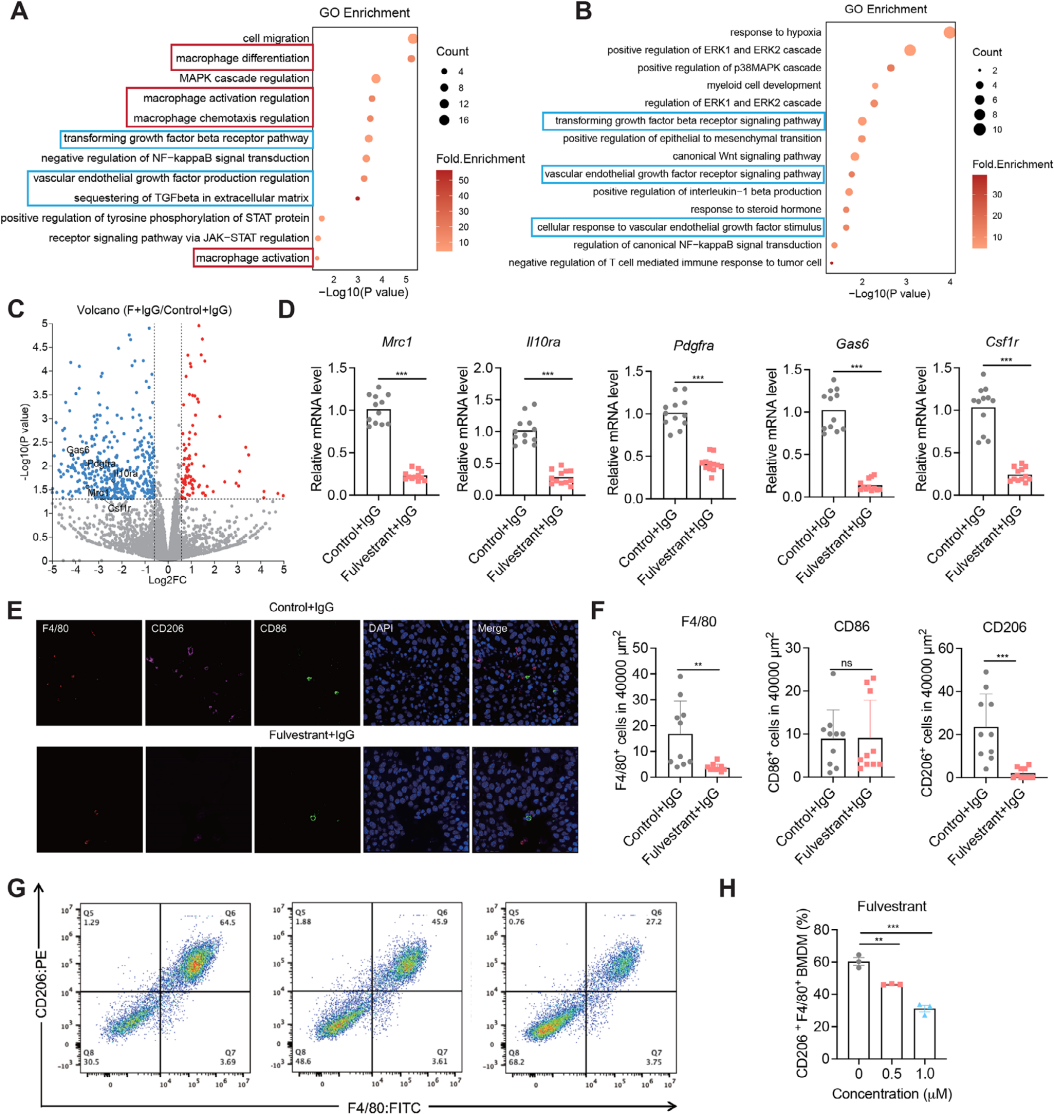
Fulvestrant remodels the tumor immune microenvironment by targeting M2‐like macrophages. A,B) Gene ontology (GO) enrichment analysis of downregulated genes from bulk RNA‐seq in Fulvestrant + IgG tumors versus Control + IgG and in Fulvestrant + anti‐PDL1 tumors compared with Control + anti‐PDL1 tumors. Key suppressed pathways include TGFβ signaling, VEGF signaling (blue rectangle), macrophage activation and chemotaxis pathways (red rectangle). C) Volcano plot showing differentially expressed genes in tumors treated with Fulvestrant + IgG compared to Control + IgG. M2 macrophage–associated genes such as *Mrc1*, *Il10ra*, *Pdgfra*, *Gas6*, and *Csf1r* are significantly downregulated in the Fulvestrant group. D) qPCR validation of *Mrc1*, *Il10ra*, *Pdgfra*, *Gas6*, and *Csf1r* in tumors treated with Fulvestrant + IgG. n = 4 mice per group; experiments were independently repeated three times. E,F) Immunofluorescence staining quantification of CD206, F4/80, and CD86 cells in tumor tissues. Fulvestrant treatment significantly reduced CD206 macrophages (M2‐like) while having significant effects on CD86 cells. n.s., not significant; ^**^ *p*< 0.01; ^***^ *p* <0.001 by unpaired t‐test. G,H) Flow cytometry analysis of bone marrow–derived macrophages (BMDMs) polarized toward the M2 phenotype in vitro in the presence or absence of Fulvestrant. All data are presented as mean ± SEM. Statistical comparisons were performed using an unpaired *t*‐test or one‐way ANOVA as appropriate.

Next, we performed transcriptomic analysis in tumors treated with Fulvestrant + IgG or Control + IgG and Fulvestrant + anti‐PDL1 or Control + anti‐PDL1. Bulk RNA‐sequencing revealed that Fulvestrant significantly downregulated multiple genes associated with immunosuppressive macrophages. Gene ontology (GO) enrichment analysis identified suppression of pathways involved in macrophage activation, chemotaxis, and differentiation, as well as signaling pathways associated with stromal and immune suppression, such as the transforming growth factor beta (TGF‐β) receptor pathway and vascular endothelial growth factor (VEGF) signaling (**Figure** **6**A,B). Notably, both TGF‐β and VEGF signaling are known to promote M2 macrophage polarization and to contribute to CD8⁺ T cell exclusion and resistance to immunotherapy.^[^ ^36^, ^37^, ^38^, ^39^ ^]^ Consistent with these findings, qPCR analysis confirmed that Fulvestrant downregulated the expression of key M2‐associated genes, including *Mrc1*, *Il10ra*, *Pdgfra*, *Gas6*, and *Csf1r* (Figure 6C,D). Thus, the observed suppression of these pathways suggests that Fulvestrant may enhance antitumor immunity by targeting M2‐associated signaling axes and facilitating T cell infiltration.

Consistent with these transcriptomic findings, immunofluorescence (IF) staining confirmed a significant reduction in F4/80⁺ total macrophages and CD206⁺ macrophages (M2‐like) in Fulvestrant‐treated tumors compared to controls (Figure 6E,F). The numbers of CD86⁺ macrophages (M1‐like) remained largely unchanged, indicating a selective depletion or reprogramming of the M2‐like immunosuppressive tumor‐associated macrophages (TAMs) population rather than a broad suppression of all macrophage subsets. Importantly, this reduction in M2‐like macrophages was accompanied by a marked increase in CD8⁺ T cell infiltration, as demonstrated in single‐cell RNA‐seq analysis, flow cytometry, and IF staining (Figure 5), suggesting that Fulvestrant relieves macrophage‐mediated suppression and facilitates cytotoxic T cell entry into the tumor microenvironment. To functionally verify whether Fulvestrant directly regulates macrophage polarization, we established an in vitro bone marrow–derived macrophage (BMDM) model. Mouse BMDMs were induced toward the M2 phenotype in the presence or absence of Fulvestrant. Flow cytometry analysis showed that Fulvestrant significantly reduced the proportion of F4/80^+^/CD206⁺ M2‐like macrophages in a dose‐dependent manner (Figure 6G,H). This finding demonstrates that Fulvestrant can directly inhibit M2 macrophage polarization, consistent with the transcriptional suppression of M2‐related genes observed in vivo.

Taken together, these findings demonstrate that Fulvestrant exerts its antitumor effects by targeting M2‐like macrophages and suppressing TGF‐β and VEGF signaling, thereby remodeling the immunosuppressive tumor microenvironment and promoting CD8⁺ T cell infiltration. This macrophage‐dependent immune modulation likely contributes to the synergistic effect of Fulvestrant when combined with ICB therapy.

## 3 Discussion

This study uncovers a vitiligo‐associated gene signature (VGS) with strong potential as both a prognostic and predictive biomarker in cancer immunotherapy. Building on the shared immune features between vitiligo and immunotherapy‐responsive tumors—including elevated CD8⁺ T cell infiltration, IFN‐γ pathway activation, antigen presentation upregulation, and chemokine‐mediated T cell recruitment—we hypothesized that immune gene programs active in vitiligo may serve as indicators of a T cell–inflamed tumor microenvironment (TME). Our findings support this hypothesis, showing that high VGS expression is associated with prolonged survival and better immunotherapy responses across multiple independent melanoma cohorts. Importantly, the predictive value of VGS was validated in both non‐ICB‐treated and ICB‐treated melanoma cohorts. In all datasets, patients with high VGS expression exhibited significantly longer median survival times and lower hazard ratios compared to low‐VGS patients. These consistent results across distinct clinical contexts highlight the robustness of VGS as a biomarker. Moreover, multivariate Cox regression analyses adjusting for known clinical covariates, such as age, gender, and tumor stage, confirmed that VGS retained its independent prognostic significance. This reinforces the potential of VGS as a clinically applicable tool for risk stratification in melanoma.

From a translational perspective, the integration of VGS into the existing immunotherapy biomarker framework could provide added value. Currently, PD‐L1 expression and TMB are among the most commonly used biomarkers for selecting patients for ICB therapies. However, both have limitations. PD‐L1 expression can be spatially heterogeneous and dynamically regulated, while TMB does not always correlate with immune activation or therapeutic benefit. In contrast, VGS reflects downstream immune activity and the functional state of the TME. Thus, combining VGS with PD‐L1 or TMB could enhance patient stratification by providing a more comprehensive assessment of tumor immunogenicity and immune engagement. Future clinical studies should explore the additive or synergistic predictive performance of these biomarkers in diverse tumor types and therapeutic settings.

In addition to its biomarker potential, we examined how pharmacologic intervention could reshape the tumor immune landscape and sensitize tumors to ICB. Specifically, we evaluated the immunomodulatory properties of Fulvestrant, a clinically approved SERD used in hormone receptor–positive breast cancer. Our transcriptomic and immunophenotypic analyses demonstrated that Fulvestrant substantially alters the TME by targeting TAMs, particularly the M2‐like immunosuppressive subset. Bulk RNA sequencing revealed that Fulvestrant downregulated multiple M2 macrophage–associated genes, including *Mrc1*, *Csf1r*, *Pdgfra*, *Gas6*, and *Il10ra*. GO analysis further highlighted suppression of macrophage activation, chemotaxis, and differentiation pathways, as well as stromal and immune suppressive signaling cascades such as TGF‐β and VEGF signaling. These pathways are known to promote M2 polarization and contribute to T cell exclusion and resistance to immunotherapy. By inhibiting these signaling axes, Fulvestrant likely creates a more permissive environment for T cell infiltration and activation.

Consistent with transcriptomic findings, flow cytometry and immunofluorescence staining confirmed a marked reduction in F4/80⁺ total macrophages and CD206⁺ M2 macrophages in Fulvestrant‐treated tumors, while the overall numbers of CD86⁺ macrophages remained unchanged. This selective depletion or reprogramming of M2 TAMs supports a mechanism in which Fulvestrant mitigates immunosuppressive myeloid influence without broadly suppressing macrophage populations. Notably, this macrophage‐targeted effect coincided with enhanced CD8⁺ T cell infiltration, as evidenced by single‐cell RNA sequencing, flow cytometry, and immunofluorescence analyses, collectively supporting a model of immune reprogramming and cytotoxic T cell recruitment. To further evaluate whether Fulvestrant acts directly on tumor cells or indirectly via the immune microenvironment, we performed a series of in vitro assays in 4T1, B16‐F10, and CT26 cell lines. Drug sensitivity curves, crystal violet staining, colony formation, and Transwell migration assays all showed that Fulvestrant did not significantly impair tumor cell viability, proliferation, or migration. These results suggest that Fulvestrant's antitumor effects are not attributable to direct cytotoxicity but are instead mediated by modulation of the tumor immune landscape.

While these findings highlight Fulvestrant's potential as an immune modulator, several limitations should be considered. Most notably, the preclinical dosing regimen used in our study—150 mg kg^−1^ every three days via intrahypodermal injection—exceeds the clinically approved human dosing of 500 mg intramuscularly once monthly.^[^ ^40^ ^]^ Recent studies suggest that lower doses in mice (e.g., 25 mg kg^−1^) may provide similar pharmacodynamic effects, indicating that excessive dosing may not be necessary to achieve immune modulation.^[^ ^41^ ^]^ Future studies should aim to optimize Fulvestrant dosing regimens, vehicle formulations, and administration routes in murine models to better reflect clinical pharmacokinetics. Moreover, long‐term evaluations of safety, sustained efficacy, and adverse effects are needed to ensure that Fulvestrant‐based immunomodulation is both effective and tolerable in combination with ICB. Mechanistically, while we identified key gene expression changes and immune population shifts associated with Fulvestrant treatment, the precise intracellular pathways and signaling nodes mediating its effects on macrophage polarization remain to be elucidated.

Taken together, our study provides new insights into the predictive potential of the vitiligo gene signature and the immunomodulatory effects of Fulvestrant. VGS offers a biologically informed and clinically relevant biomarker that may enhance the precision of immunotherapy stratification. Concurrently, Fulvestrant emerges as a promising adjunctive agent capable of remodeling the tumor microenvironment and potentiating immune responses. These findings lay the groundwork for future translational efforts to integrate immune biomarkers with rationally designed combination therapies, ultimately improving outcomes for patients receiving cancer immunotherapy.

## 4 Experimental Section

### Weighted Gene Co‐Expression Network Analysis (WGCNA) Method

Network analysis of large datasets is an essential tool for identifying key molecular pathways and potential new drug targets. Co‐expression modules in networks are linked to disease processes, in which the most centrally connected genes are highly enriched for key drivers that play prominent roles in disease pathogenesis. To facilitate the identification of key modules and genes related to a specific clinical trait in a disease, Zhang et al. proposed the Weighted Gene Co‐expression Network Analysis (WGCNA) method. This method assumes that the patient's gene regulatory network follows a scale‐free topology, approximated by a power‐law distribution. To construct this scale‐free network, the correlation coefficients between genes were raised to a soft‐thresholding power β, which were selected based on the criterion of achieving a scale‐free topology fit index (R^2^) >0.85. In the analysis, a soft‐thresholding power of 10 was used. After adjacency matrix construction and transformation into a topological overlap matrix (TOM), genes were hierarchically clustered into modules. Modules with highly similar eigengenes were merged using a merge cut height of 0.25. The first principal component of each module was defined as the module eigengene (ME). In the study, the types of vitiligo patients—healthy controls, lesional skin, perilesional skin, and non‐depigmented skin—were used as the “trait,” labeled as 0, 1, 2, and 3, respectively. Pearson correlation analysis was then performed between these traits and MEs to identify modules most significantly associated with the clinical condition. Genes within these key modules were considered for downstream analysis. WGCNA analysis was performed using the “WGCNA” package^[^ ^42^ ^]^ in R.

### Vitiligo Gene Signature Identification

The WGCNA method identified two modules that were most related to the different stages of vitiligo, namely the blue and dark orange modules (Figure S1B, Supporting Information). Meanwhile, the limma package was used to obtain the differentially expressed genes (DEGs) between lesional and healthy tissue. Among these DEGs, there were 226 overlapping genes with the WGCNA modules. The uniCox analysis was then used to identify genes related to the survival of melanoma patients among these 226 genes, and found 18 genes with a *p*‐value less than 0.05. Notably, 10 of these genes had the same expression pattern as that of vitiligo, namely GP1BA, ANKS4B, CCDC87, CA8, HLA‐DOB, RHEBL1, NLRP7, GZMH, HERPUD1, and MAP2K1, and these 10 genes were designated as the vitiligo gene signature (VGS) (Figure 1B; Figure S1A, Supporting Information).

### Clustering

The expression profiles of the ten vitiligo‐related genes were utilized to identify melanoma subtypes in both the GEO cohort (GSE65904) and the TCGA cohort. All tumor samples were grouped into different subtypes (k = 2) using the consensus clustering method (shown in Figure S1F,H, Supporting Information). As the TCGA cohort contained more clinical information, further analysis was performed in this cohort, which included immune cell state analysis and signaling pathway analysis based on GSVA.^[^ ^43^ ^]^

### Vitiligo Score (VS)

The Vitiligo Score was calculated as the unweighted sum of the expression levels of ten genes: GP1BA, ANKS4B, CCDC87, CA8, HLA‐DOB, RHEBL1, NLRP7, GZMH, HERPUD1, and MAP2K1. Patients were classified into high and low Vitiligo Score (VS) groups based on the median score. Survival differences between the groups were assessed using Kaplan–Meier (KM) analysis, with univariate Cox proportional hazards models applied to evaluate statistical significance. This analysis was conducted on three cohorts, namely two melanoma cohorts (GSE65904 and TCGA) and one immunotherapy cohort (IMvigor210). Additionally, the expression of checkpoint genes, the 22 immune cell states, and the difference in tumor mutation burden (TMB) were analyzed in the TCGA cohort.

### Immune Cell Analysis in Two Subtypes

To determine the proportions of immune cells in melanoma cancer samples, the CIBERSORT algorithm was utilized, which provides sensitive and specific discrimination of 22 human immune cell types. CIBERSORT is a deconvolution algorithm that employs a signature of 547 genes as a minimal representation for each cell type.^[^ ^44^ ^]^ Using support vector regression, cell types were inferred based on gene‐expression values. The expression values of checkpoint genes were also compared between the low and high Vitiligo Score (VS) groups and a *t*‐test was performed for statistical analysis.

### Cell Lines

The B16F10, B16‐F10‐OVA mouse melanoma cells, CT26 mouse colon carcinoma cells, and 4T1 mouse breast carcinoma cells were purchased from the American Type Culture Collection (ATCC; Manassas, VA, USA) and their identities were verified using the short tandem repeat (STR) method. The cells were cultured in DMEM (Gibco, C11995500BT) supplemented with 10% FBS (Gibco, A5256701), and were regularly checked for mycoplasma contamination using a universal detection kit (Meilunbio, FM311‐01).

### Tumors Growth in Mice

All animal experiments were approved by the Qingdao University Institutional Animal Use and Care Committee (QDU‐AEC‐2022083). C57BL/6JNifdc and BALB/c mice were purchased from Charles River Laboratory (Beijing Vital River Laboratory Animal Technology Co., Ltd.). Mice were housed at an ambient temperature of 21 °C, with a humidity of 40–70%, and a light cycle of 12 h on/12 h off set from 7 am to 7 pm. Before tumor cells were injected, age‐matched 6–8‐week‐old mice were shaved at the flank. In experiments involving PD‐L1‐neutralizing antibodies, 6–8‐week‐old syngeneic C57BL/6JNifdc or BALB/c mice were inoculated subcutaneously with B16‐F10‐luc cells (1 × 10^5^ cells per mouse^−1^), 4T1‐luc cells (2 × 10^5^ cells per mouse^−1^), CT26‐luc cells (1 × 10^5^ cells per mouse^−1^) or B16‐F10‐OVA cells (1 × 10^5^ cells per mouse^−1^) into the shaved flank subcutaneously, respectively.

### Drug Information and Treatments

The small‐molecule inhibitors Fulvestrant (T2146), Cobicistat (T6246), and Motolimod (T6898) were purchased from TargetMol, and were suspended in 10% 2‐hydroxypropyl‐β‐cyclodextri(H108813, Aladdin) and 4% DMSO (PHR1309, Sigma) in water. When the tumor growth was measurable (6 days after the injection), C57BL/6JNifdc or BALB/c tumor‐bearing mice were assigned into 4 groups that received the vehicle orally (100 mL of 10% 2‐hydroxypropyl‐β‐cyclodextrin /4% DMSO every day, po.) or Fulvestrant (150 mg kg^−1^ in a final formulation in 10% 2‐hydroxypropyl‐β‐cyclodextrin/4% DMSO every 3 days, ih.) or Cobicistat (20 mg kg^−1^ in a final formulation in 10% 2‐hydroxypropyl‐β‐cyclodextrin/4% DMSO every day, po.) or Motolimod (2 mg kg^−1^ in a final formulation in 10% 2‐hydroxypropyl‐β‐cyclodextrin/4% DMSO every 2 days, ih.) for 16 days. For combination therapy, inhibitors treatment was administered for 5 days before combining with or i.p. injection of either anti‐PD‐L1 antibody (150 µg every 3 days, a total of 4 times: clone 10F.9G2, BE0101, BioXcell) or IgG2b isotype control antibody (150 µg every 3 days, a total of 4 times; clone LTF2, BE0090, BioXcell). Anti‐PD‐L1 monoclonal antibodies or human IgG2b isotype control were injected (intraperitoneally) on days 6, 9, 12, and 15. At least six mice per treatment were included. Importantly, randomization and blinding were not employed in these experiments. While this approach is common in exploratory studies, the absence of these controls can introduce selection and observer biases, potentially affecting the objectivity of tumor volume assessments and other outcome measures. Randomization helps distribute both known and unknown confounding factors evenly across treatment groups, reducing the risk of selection bias. Blinding minimizes the potential for observer bias, especially in subjective measurements like tumor size assessments. Future studies will incorporate randomization and blinding protocols to enhance the rigor and reproducibility of the findings. Tumor size was measured using a caliper and calculated using the formula volume = (length)(width)^2^/2. The endpoint was defined as the time at which a progressively growing tumor reached 1.5 cm in its longest dimension or 2000 mm^3^ in diameter. Mice were also euthanized when they experienced open skin lesions, lost more than 15% of their total body weight, or failed to thrive.

### Flow Cytometry

Tumors were dissected from mice and mechanically dissociated and digested in 1640 medium supplemented with 3 mg mL^−1^ collagenase II and 100 µg mL^−1^ DNase I (Roche, 11284932001) with the addition of 1 mm sodium pyruvate. The digestion was performed at 37 °C for 60 min. The digestion was terminated by adding 1640 medium (Gibco, C11875500BT) containing 10% FBS, and the digested material was filtered through a 70 µm cell strainer to obtain single‐cell suspensions for flow cytometry analysis. A total of 1 × 10^6 cells were incubated with TruStain FcX PLUS (anti‐mouse CD16/32) antibody (clone: S17011E, Biolegend, 156604) for 10 min to prevent nonspecific Fc receptor‐mediated antibody staining. The single‐cell suspensions were then incubated with a mixture of fluorescently labeled monoclonal antibodies at 4 °C for 15 min. After surface staining, the cells were washed twice and stained with 7‐AAD (Biolegend, 420403) for viability assessment before direct analysis. Flow cytometry sample acquisition was performed on a CytoFLEX S flow cytometer (Beckman Coulter Life Sciences, Krefeld, Germany). Data were analyzed using FlowJo software. The antibodies used are CD45 FITC (clone:30‐F11, Biolegend, 103108), CD4 PE (GK1.5, Biolegend, 100407), CD8 APC (53‐6.7, Biolegend, 100712), and 7‐AAD.

### scRNA‐seq Profiling and Data Analysis

A public scRNA‐seq dataset (GSE213733) and a public spatial transcriptome dataset (GSE206245) were used for analysis. For the scRNA‐seq dataset analysis, 1,2741 cells passed the quality control criteria, which included removing genes expressed in less than three cells, setting a low and high cutoff for the number of genes expressed per cell (>500 and <5000, respectively), requiring UMI counts greater than 500, and limiting the percent of mitochondrial‐DNA derived gene‐expression to <25%. For dimensionality reduction and data visualization, the LogNormalize method in the “Normalization” function of the Seurat package^[^ ^45^ ^]^ was first used to calculate the expression profiles. Next, principal component analysis (PCA) was performed on the normalized expression profiles, with the top‐15 principal components (PCs) used for t‐SNE and UMAP analysis. A total of 19 cell clusters were identified using the weighted Shared Nearest Neighbor (SNN) graph‐based clustering method.^[^ ^45^ ^]^ The 19 clusters were manually annotated with established cell type markers (Table S1, Supporting Information) and obtained 11 cell types.

### Ethics Statement

The analysis of human breast cancer biopsies study was reviewed and approved by the Ethics Committee Medical College of Qingdao University (Number: QDU‐HEC‐2022046). All donors signed the written informed consent for sample collection and data analysis. Before signing the consent, the necessary information including the goals and related experimental procedures for the study were provided to the donors.

### qRT–PCR of Gene Expression

Total RNA was extracted from tumors (≈200–300 mm^3^ in volume) from tumor‐bearing mice using the RNeasy Mini Kit (Qiagen) according to the manufacturer's instructions. RNA was subjected to cDNA synthesis with random hexamer primers using Superscript II reverse transcriptase (Invitrogen, 18080044). Real‐time quantitative RT–PCR (qRT–PCR) was performed using a QuantiTest SYBR Green PCR master mix kit (Mei5bio, MF797). Primers used in experiments are listed here:

*Gp1ba*‐F: 5′‐CATTCCCAGCACACTTGTAGT‐3′

*Gp1ba*‐R: 5′‐TGAGGTGAGTGAAATGCACCA‐3′

*Herpud1*‐F: 5′‐GCAGTTGGAGTGTGAGTCG‐3′

*Herpud1*‐R: 5′‐TCTGTGGATTCAGCACCCTTT‐3′

*Map2k1*‐F: 5′‐AAGGTGGGGGAACTGAAGGAT‐3′

*Map2k1*‐R: 5′‐CGGATTGCGGGTTTGATCTC‐3′

*H2‐Ob*‐F: 5′‐AGGCGGACTGTTACTTCACC‐3′

*H2‐Ob*‐R: 5′‐ATCCAGGCGTTTGTTCCACTG‐3′

*Gzmb*‐F: 5′‐CCACTCTCGACCCTACATGG‐3′

*Gzmb*‐R: 5′‐GGCCCCCAAAGTGACATTTATT‐3′

*Gzmc*‐F: 5′‐GCAGAGGAGATAATCGGAGGC‐3′

*Gzmc*‐R: 5′‐GCACGAATTTGTCTCGAACCA‐3′

*Csf1r‐*F: GGACCTACCGTTGTACCGAG

*Csf1r*‐R: CAAGAGTGGGCCGGATCTTT

*Gas6*‐F: CCGCGCCTACCAAGTCTTC

*Gas6*‐R: CGGGGTCGTTCTCGAACAC

*Pdgfra*‐F: GGAGACTCAAGTAACCTTGCAC

*Pdgfra*‐R: TCAGTTCTGACGTTGCTTTCAA

*Il10ra*‐F: GCCCTTCCTATGTGTGGTTTG

*Il10ra*‐R: TTGAGTTTCCGTACTGTTTGAGG

*Mrc1*‐F: CTCTGTTCAGCTATTGGACGC

*Mrc1*‐R: TGGCACTCCCAAACATAATTTGA

*β‐actin*‐F: 5′‐GAAATCGTGCGTGACATCAAAG‐3′

*β‐actin*‐R: 5′‐TGTAGTTTCATGGATGCCACAG‐3′

### Co‐Culture of Cancer Cells and T Cells for T Cell Cytotoxicity Assay

To evaluate the chemotactic response of T cells within the splenic immune cell population, a Transwell migration assay was conducted using 24‐well inserts with 5 µm pore polycarbonate membranes (Corning). Specifically, Pmel‐1 T cells were used for migration toward B16‐F10 cells, and OT‐1 T cells were used for B16‐F10‐OVA targets to ensure antigen specificity. Tumor cells (B16‐F10 or B16‐F10‐OVA) were seeded in the lower chambers at a density of 1 × 10⁵ cells per well. Spleens were harvested from Pmel‐1 (Jackson Laboratory, Strain No: 005023) or OT‐1 (Cavens, Strain No: D000053) transgenic mice, which had been immunized twice with tumor cells, and single‐cell suspensions were prepared by mechanical dissociation followed by red blood cell lysis. The resulting splenocyte suspensions were resuspended in complete splenic lymphocyte culture medium (CM‐M153, Procell) and adjusted to a concentration of 1 × 10⁶ cells per 100 µL. This suspension was added to the upper chamber of the transwell inserts. Following a 12‐h incubation with Fulvestrant, Cobicistat, Motolimod, or vehicle control at 37 °C in a humidified 5% CO_2_ incubator, migrated cells in the lower chamber were collected. The proportion of CD8⁺ T cells among the migrated cells was determined by flow cytometry using CD8‐specific antibody staining. All conditions were run in triplicate, and each experiment was independently repeated at least three times.

### Immunofluorescence and Immunohistochemistry

For immunofluorescence analysis, tumors from mice were fixed in 10% neutral‐buffered formalin, embedded into paraffin, sectioned and then mounted onto slices. They were then stained by standard procedures using antibodies against mouse (or human) CD45 or CD8a or CD4.

### Cell Viability Assay (CCK‐8 Assay)

Cells in the logarithmic growth phase were harvested and seeded into 96‐well plates at a density of 1 × 10⁴ cells per 100 µL per well. After a 24‐h preincubation at 37 °C in a humidified atmosphere with 5% CO_2_, the medium was replaced with complete culture medium containing varying concentrations of the indicated compounds. After 72 h of drug exposure, 10 µL of CCK‐8 reagent (Dojindo, Japan) was added to each well, and plates were incubated for an additional 2 h. Absorbance was measured at 450 nm using a microplate reader (BioTek). All experiments were performed in triplicate.

### Colony Formation Assay

Cells were digested using trypsin and resuspended in complete medium as a single‐cell suspension. The cell concentration was adjusted to 1000 cells mL^−1^, and 200 cells were seeded into each well of a 12‐well plate. After 24 h of adhesion, the culture medium was replaced with complete medium containing Fulvestrant at 0, 0.5, 2.5, or 5 µm. Cells were cultured for 10 days, with medium changes every 3 days. At the end of the incubation period, cells were washed with PBS (Gibco, 10099‐141) and fixed with 4% paraformaldehyde (1 mL well^−1^) for 30–60 min at room temperature. After fixation, cells were washed again and stained with 0.1% crystal violet solution for 15 min. Colonies were photographed, and absorbance was quantified at 590 nm using a microplate reader. Experiments were repeated three times.

### Transwell Migration Assay

CT26, 4T1, and B16‐F10 cells were serum‐starved for 24 h prior to the assay. CT26 and 4T1 cells were resuspended at 1 × 10⁵ cells mL^−1^, and B16‐F10 cells at 2 × 10⁵ cells mL^−1^. A 100 µL suspension was seeded into the upper chambers of transwell inserts (8 µm pore size; Corning, USA). The lower chambers were filled with 1 mL of complete medium containing 10% FBS (Gibco, A5256701) and Fulvestrant at concentrations of 0, 0.5, 2.5, or 5 µm. After 48 h of incubation at 37 °C, non‐migrated cells on the upper surface were gently removed with a cotton swab. Migrated cells on the lower surface were fixed with 4% paraformaldehyde for 10 min, washed with PBS, and stained with 0.5% crystal violet for 15 min. After three additional washes with PBS, cells were air‐dried and imaged under a microscope (200× magnification). The number of migrated cells was quantified by counting five random fields per well.

### Isolation and Culture of BMDMs

Bone marrow‐derived macrophages (BMDMs) were isolated from the femurs and tibias of 6–8‐week‐old male and female C57BL/6J mice using a previously described method.^[^ ^46^ ^]^ The cells were maintained in DMEM supplemented with 10% FBS, 1% penicillin/streptomycin (P/S), and 100 ng mL^−1^ macrophage colony‐stimulating factor (M‐CSF1; Novoprotein, CB34). After 24 h of culture, non‐adherent cells were removed. Following 7 days of differentiation, floating cells were discarded, and adherent macrophages were harvested and seeded into multiwell plates for subsequent experiments.

### Macrophage Polarization and Treatment

For M2 polarization, macrophages were incubated for 24 h in polarization medium containing 25 ng mL^−1^ M‐CSF1, 20 ng mL^−1^ interleukin‐4 (IL‐4; Sino Biological, 51084‐MNAE), and 20 ng mL^−1^ interleukin‐13 (IL‐13; Novoprotein, CX56). Fulvestrant was then added to the culture. Macrophage differentiation and polarization were confirmed by flow cytometry, with successful differentiation defined by the presence of CD206 (BioLegend, 141705) and F4/80 (BioLegend, 123107) double‐positive cells.

## Conflict of Interest

The authors declare no conflict of interest.

## Author Contributions

J.Z., L.H., S.B., and W.K. contributed equally to this work. J.Z., Z.X., and S.Z. designed experiments and discussed the results. J.Z., W.K., and S.Z. contributed to all stages of manuscript preparation and editing. Material preparation, data collection, and analysis were performed by J.Z., L.H., W.K., S.B., W.F., Y.L., Z.X., P.S., and S.Z. supervised all the teams. All authors read and approved the final manuscript.

## Supporting information



Supporting Information

Supplemental Figures

## Data Availability

The data that support the findings of this study are available on request from the corresponding author. The data are not publicly available due to privacy or ethical restrictions.
