# Supplementary material for: Vitiligo Signature‐Based Drug Screening Identifies Fulvestrant as a Novel Immunotherapy Combination Strategy
Source: Adv Sci (Weinh). 2025 Sep 20;12(44):e03979. doi: 10.1002/advs.202503979 (PMC12667482; doi:10.1002/advs.202503979)
Supplement: Supplementary file 1 — Supporting Information [file ADVS-12-e03979-s002.docx]

Supplementary Materials

Figure S1 to S10

**Figure S1. Vitiligo signature was identified by analyzing the vitiligo cohort and the melanoma cohort at the same time.** A, the flow chart of identifying vitiligo gene signature genes (VGS) in GSE65127 cohort. B, Module-trait relationships using Pearson correlation coefficient (Student asymptotic p-value). “group” varible was the types of vitiligo patients, including healthy controls, lesional skin, perilesional skin, and non-depigmented skin, which were labeled as 0,1,2,3, respec- tively. Detailed instruction of WGCNA analysis was in “Method” part. C, the number of overlapping genes between DEGs and WGCNA analysis. D-H, Consensus clustering based on vitiligo signature genes in two different melanoma cohorts, including GSE65904 (D, E, G) and TCGA cohort (F, H). D, Consensus matrices of the GSE65904 cohort for k=2. E, the survival plot for the two clusters in GSE65904 cohort using Kaplan-Meier curves including 52 cases in cluster 1, 158 cases in cluster 2. The cluster 2 showed significantly better overall survival than the cluster 1. (p=0.002, Log-rank test). F, the Consensus matrices of the TCGA cohort for k=2. G, the heatmap showed the expression of VGS in GSE65904 cohort. H, the heatmap showed the expression of VGS in TCGA cohort.

**Figure S2. Two clusters have differential immune states.** A, the state of different types of immune cells between cluster1 and cluster2, and statistical significance was assessed by Wilcoxon test (*p < 0.05, **p < 0.01, ***p < 0.001 and ****p < 0.0001). B, the signaling pathway upregulated in cluster1. C, the signaling pathway upregulated in cluster2.

**Figure S3. Vitiligo score was significantly related to immunity.** A, the process of constructing the vitiligo score (VS). B, the VS comparison between the cluster1 and cluster2 (Wilcoxon test). C, the expres- sion of check point genes between high VS group and low VS group, and the statistical significance was assessed by Wilcoxon test (*p < 0.05, **p < 0.01, ***p < 0.001 and ****p < 0.0001). D, the expression of genes of T cell-inflamed GEP, and the statistical significance was assessed by Wilcoxon test (*p < 0.05, **p < 0.01, ***p < 0.001 and ****p < 0.0001). E, the mutations in the high VS group. F, the mutations in the low VS group. G, the comparison of TMB score between high and low VS groups. H, the comparison of immune cells between high and low VS group.

**Figure S4. Responder patients in immunotherapy exhibited higher expression of VGS compared with non-responder patients.** A-C, the progression-free survival of melanoma patients stratified by the expression of ANKS4B (A), RHEBL1 (B) and GZMH (C). Log-rank test. D, the gene expression of HLA-DOB, GZMH, RHEBL1, HERPUD1, NLRP7, and MAP2K1 in complete response (CR) and stable disease (SD) patients. Box- plots show the expression of six VGS genes in immunotherapy responders (R) versus non-responders (NR) in the Hugo et al. dataset. Expression values are shown as Log(Expression + 1). Statistical comparisons were performed using the Wilcoxon rank-sum test (p< 0.05; p< 0.01; p< 0.001; ns = not significant).

**Figure S5. The impact of the combination of Cobicistat and Selinexor with PD-L1 on tumor progression.** A, Relative T cell proportion in B16-F10 cell and B16-F10-OVA cell treated with 50nM and 500nM Cobicistat for 48h. B, Relative T cell proportion in B16-F10 cell and B16-F10-OVA cell treated with 5nM and 50nM Selinexor for 48h. C-D, Tumor sizes at day 19 (sample-paired Student’s t-test). *p < 0.05, **p< 0.01, ***p < 0.001, and ****p < 0.0001. Error bars depict SEM. E, Illustration of animal models. B16-F10 cells were injected into C57BL mice. Animals were administrated when the volumes of tumors were about 50 mm3. F, The growth of B16-F10 tumors was measured by tumor volume; volume (mm3) = [width2 (mm2) × length (mm)]/2. G, Tumor sizes at day 19 (sam- ple-paired Student’s t-test). *p< 0.05, **p < 0.01, ***p < 0.001, and ****p < 0.0001. Error bars depict SEM. H, Kaplan-Meier plots demonstrating the association between B16-F10 tumors and overall survival. Log-rank test.

**Figure S6. Fulvestrant modulates VGS gene expression in B16-F10 cells.** A–B, Gene expres- sion analysis showing the transcriptional changes of vitiligo gene signature (VGS) components in B16-F10 tumor cells following treatment with (A) Fulvestrant and (B) Motolimod. Data demonstrate the impact of each drug on VGS-related genes involved in immune regulation and potential response to immunotherapy.

**Figure S7. Dot plot showing canonical marker gene expression used to define major cell types.** The dot plot visualizes the expression patterns of representative marker genes across different cell clusters. Dot size reflects the percentage of cells expressing the gene within each cluster, and color intensity indicates the average expression level.

**Figure S8. The effects of Fulvestrant immunotherapy on immune cells.** (A-B) Representative flow cytometry plots of CD8+ cells (A) and the percentages of CD8+ cells within the CD45+ cell population (B) in CT26 xenograft tumors (n=6) after four injections of PD-L1 antibody in different treatment groups. (C-D) Representative flow cytometry plots of CD8+ cells (C) and the percentages of CD8+ cells within the CD45+ cell population (D) in 4T1 xenograft tumors (n=5) after four injections of PD-L1 antibody in different treatment groups. One-way ANOVA was used to determine statistical significance. Data are presented as mean ± SEM. *p<0.05; **p<0.01; ***p<0.001; ****p<0.0001.

**Figure S9. Fulvestrant significantly activated the antigen processing and presentation signaling pathways.** A-C, the differences of cell communication strength between E2 and E2+ICI group in CXCL, IFN-II, and MHC-I signaling pathways. After Fulvestrant perturbation, the cell communication of the above three pathways was increased for the cytotoxic T cells (CD8+ and NK cells) and myoepithelial cells.

**Figure S10. Fulvestrant does not directly impair tumor cell viability, proliferation, or migration in vitro.** A–C, bar plots showing the viability of B16-F10, 4T1, and CT26 tumor cell lines after treatment with different doses of Fulvestrant, assessed by CCK-8 assay. D-F, Dose–response curves showing the viability of B16-F10, 4T1, and CT26 tumor cell lines after treatment with Fulvestrant alone or Fulvestrant + IgG, assessed by CCK-8 assay. G, Crystal violet staining and quantification of colony formation after treatment with different concentrations of Fulvestrant in B16-F10, 4T1, and CT26 cells. No significant differences were observed. H, Transwell migration assay showing that Fulvestrant treatment (0.5, 2.5, and 5μM) does not significantly affect the migration capacity of B16-F10, 4T1, or CT26 tumor cells. Data are shown as mean ± SEM from at least three independent experiments. ns = not significant.

Tables S1 to S3:

**Table S1 The input genes for DLEPS**

| **Gene Symbol** | **Gene ID** |
| --- | --- |
| **GP1BA** | **2811** |
| **HLA-DOB** | **3112** |
| **GZMH** | **2999** |
| **HERPUD1** | **9709** |
| **MAP2K1** | **5604** |

**Table S2 The top 10 compounds based on the input genes**

| **ID** | **Name** | **CAS Number** | **Score** |
| --- | --- | --- | --- |
| **S1191** | **Fulvestrant** | **129453-61-8** | **0.480759247** |
| **S7161** | **Motolimod (VTX-2337)** | **926927-61-9** | **0.46972745** |
| **S2900** | **Cobicistat (GS-9350)** | **1004316-88-4** | **0.467910448** |
| **S7252** | **Selinexor (KPT-330)** | **1393477-72-9** | **0.459231019** |
| **S4078** | **Mefenamic Acid** | **61-68-7** | **0.45793316** |
| **S2032** | **Rebamipide** | **90098-04-7** | **0.449789098** |
| **S1048** | **Tozasertib (VX-680, MK-0457)** | **639089-54-6** | **0.44954575** |
| **S6003** | **Ataluren (PTC124)** | **775304-57-9** | **0.448977936** |
| **S2922** | **Icotinib** | **610798-31-7** | **0.444841012** |
| **S1255** | **Nepafenac** | **78281-72-8** | **0.442326411** |

**Table S3 The marker genes for 11 cell types**

| Cell type | Maker genes |  |  |  |  |  |  |
| --- | --- | --- | --- | --- | --- | --- | --- |
| Myoepithelial cells | Krt17 | Krt14 | Krt5 | Acta2 | Myl9 | Mylk | Myh11 |
| Luminal epithelial cells | Krt19 | Krt18 | Krt8 |  |  |  |  |
| Fibroblast | Col1a1 | Col1a2 | Col3a1 | Fn1 |  |  |  |
| B cells | Cd19 | Cd79a | Ms4a1 |  |  |  |  |
| Plasma cells | Ighg1 | Mzb1 | Sdc1 |  |  |  |  |
| Myeloid cells | Cd68 | Cd163 | Csf1r | Mrc1 | Tpsab1 | Ms4a2 | Cd1c |
| CD8+ T and NK cells | Cd3d | Cd3e | Cd3g | Cd2 | Nkg7 | Ncr1 |  |
| CD4+ T cells | Cd3d | Cd3e | Cd3g | Cd2 | Cd4 |  |  |
| Endothelial cells | Pecam1 | Vwf |  |  |  |  |  |
| Epithelial cells | Epcam | Krt19 | Prom1 |  |  |  |  |
| Neutrophils | Csf3r | S100a8 | Cxcl3 |  |  |  |  |
