# Supplementary material for: Vitiligo Signature‐Based Drug Screening Identifies Fulvestrant as a Novel Immunotherapy Combination Strategy
Source: Adv Sci (Weinh). 2025 Sep 20;12(44):e03979. doi: 10.1002/advs.202503979 (PMC12667482; doi:10.1002/advs.202503979)
Supplement: Supplementary file 2 — Supplemental Figures [file ADVS-12-e03979-s001.zip › advs71623-sup-0002-FigureS1.pdf]

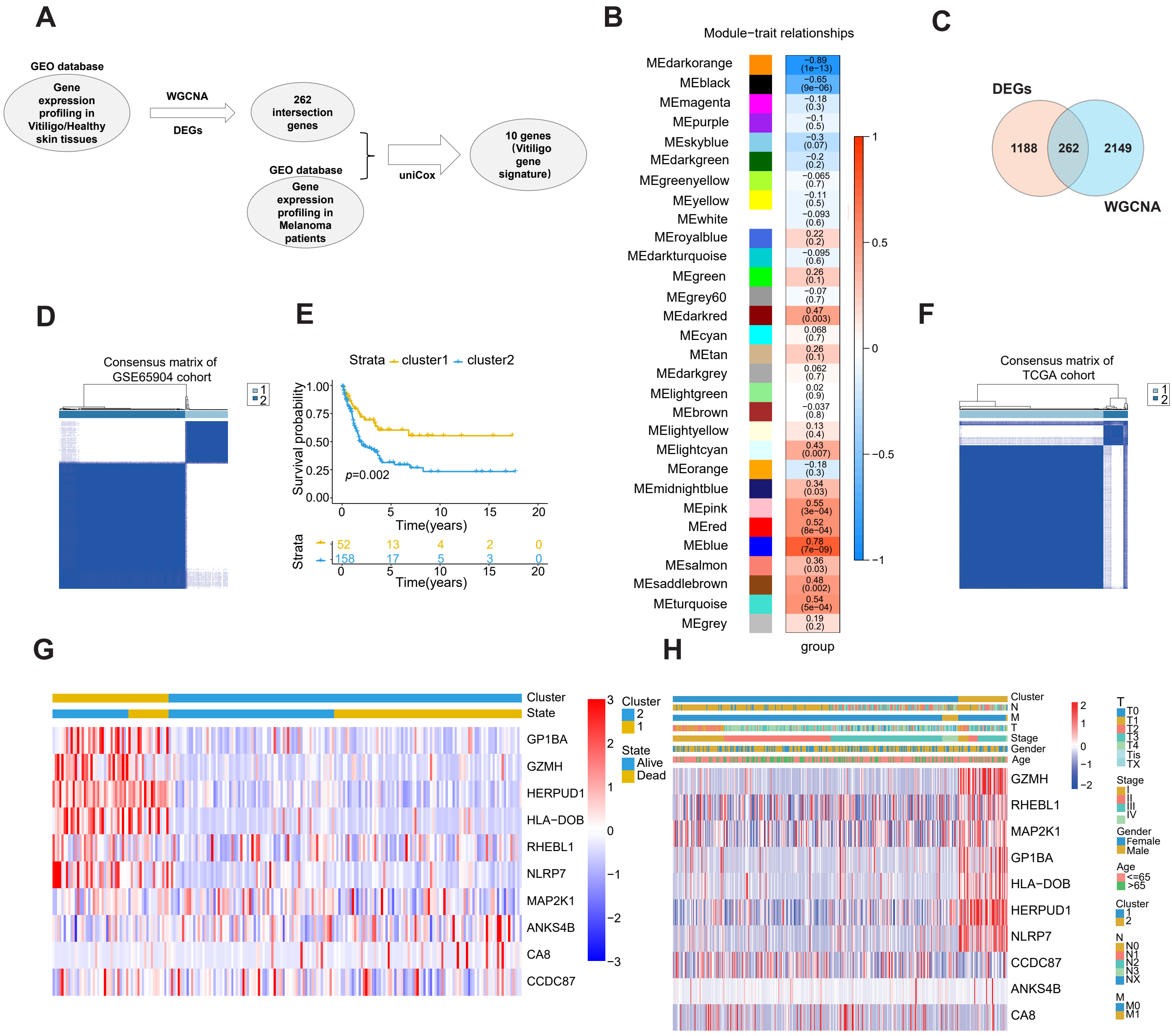

Figure S1. Vitiligo signature was identified by analyzing the vitiligo cohort and the melanoma cohort at the same time. A, the flow chart of identifying vitiligo gene signature genes (VGS) in GSE65127 cohort. B, Module-trait relationships using Pearson correlation coefficient (Student asymptotic p-value). “group” varible was the types of vitiligo patients, including healthy controls, lesional skin, perilesional skin, and non-depigmented skin, which were labeled as 0,1,2,3, respectively. Detailed instruction of WGCNA analysis was in “Method” part. C, the number of overlapping genes between DEGs and WGCNA analysis. D-H, Consensus clustering based on vitiligo signature genes in two different melanoma cohorts, including GSE65904 (D, E, G) and TCGA cohort (F, H). D, Consensus matrices of the GSE65904 cohort for k=2. E, the survival plot for the two clusters in GSE65904 cohort using Kaplan-Meier curves including 52 cases in cluster 1, 158 cases in cluster 2. The cluster 2 showed significantly better overall survival than the cluster 1. (p=0.002, Log-rank test). F, the Consensus matrices of the TCGA cohort for k=2. G, the heatmap showed the expression of VGS in GSE65904 cohort. H, the heatmap showed the expression of VGS in TCGA cohort.
