# Supplementary material for: Vitiligo Signature‐Based Drug Screening Identifies Fulvestrant as a Novel Immunotherapy Combination Strategy
Source: Adv Sci (Weinh). 2025 Sep 20;12(44):e03979. doi: 10.1002/advs.202503979 (PMC12667482; doi:10.1002/advs.202503979)
Supplement: Supplementary file 2 — Supplemental Figures [file ADVS-12-e03979-s001.zip › advs71623-sup-0003-FigureS2.pdf]

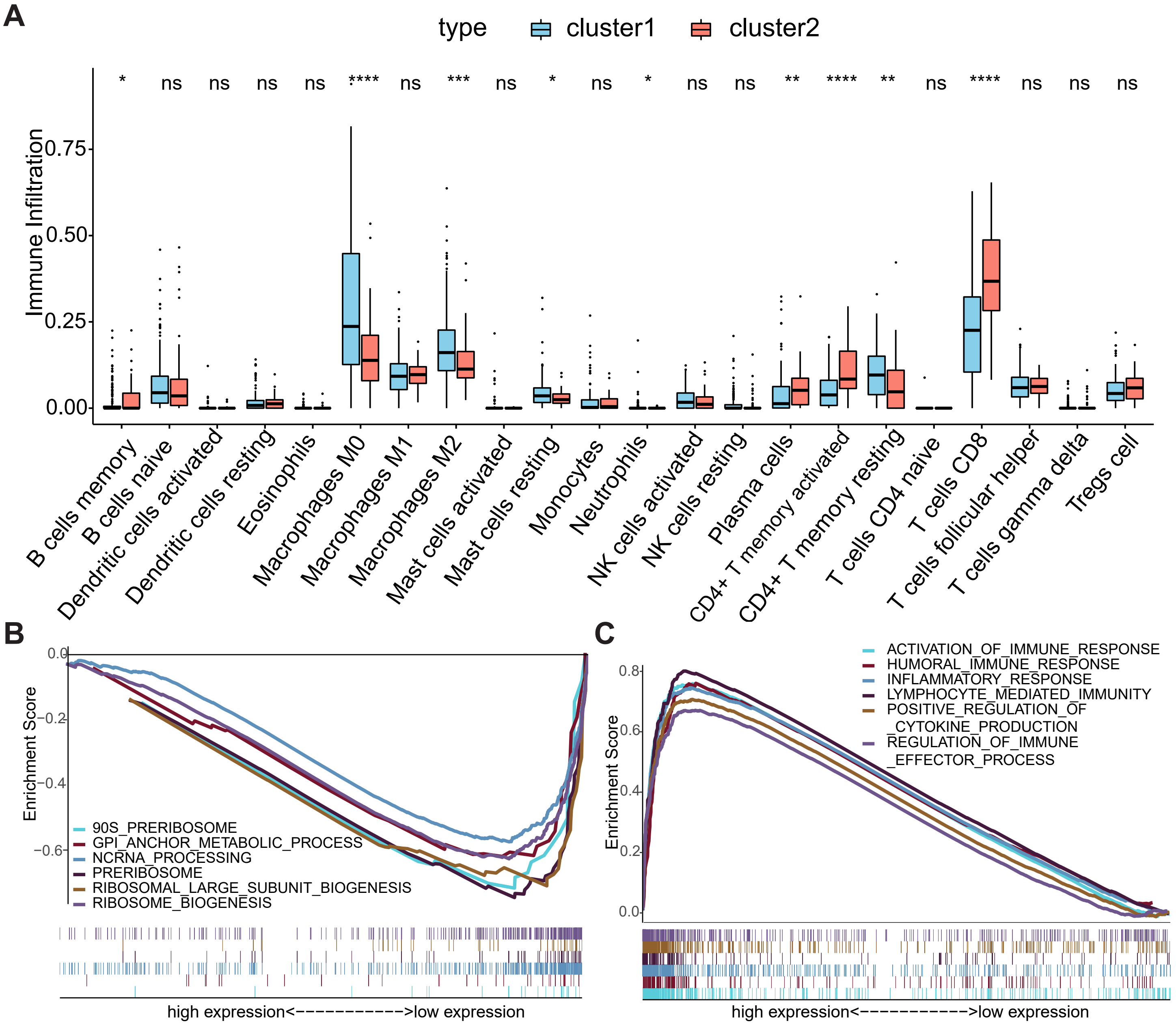

Figure S2. Two clusters have differential immune states. A, the state of different types of immune cells between cluster1 and cluster2, and statistical significance was assessed by Wilcoxon test (*$p < 0.05$, **$p < 0.01$, ***$p < 0.001$ and ****$p < 0.0001$). B, the signaling pathway upregulated in cluster1. C, the signaling pathway upregulated in cluster2.
