# Supplementary material for: Vitiligo Signature‐Based Drug Screening Identifies Fulvestrant as a Novel Immunotherapy Combination Strategy
Source: Adv Sci (Weinh). 2025 Sep 20;12(44):e03979. doi: 10.1002/advs.202503979 (PMC12667482; doi:10.1002/advs.202503979)
Supplement: Supplementary file 2 — Supplemental Figures [file ADVS-12-e03979-s001.zip › advs71623-sup-0004-FigureS3.pdf]

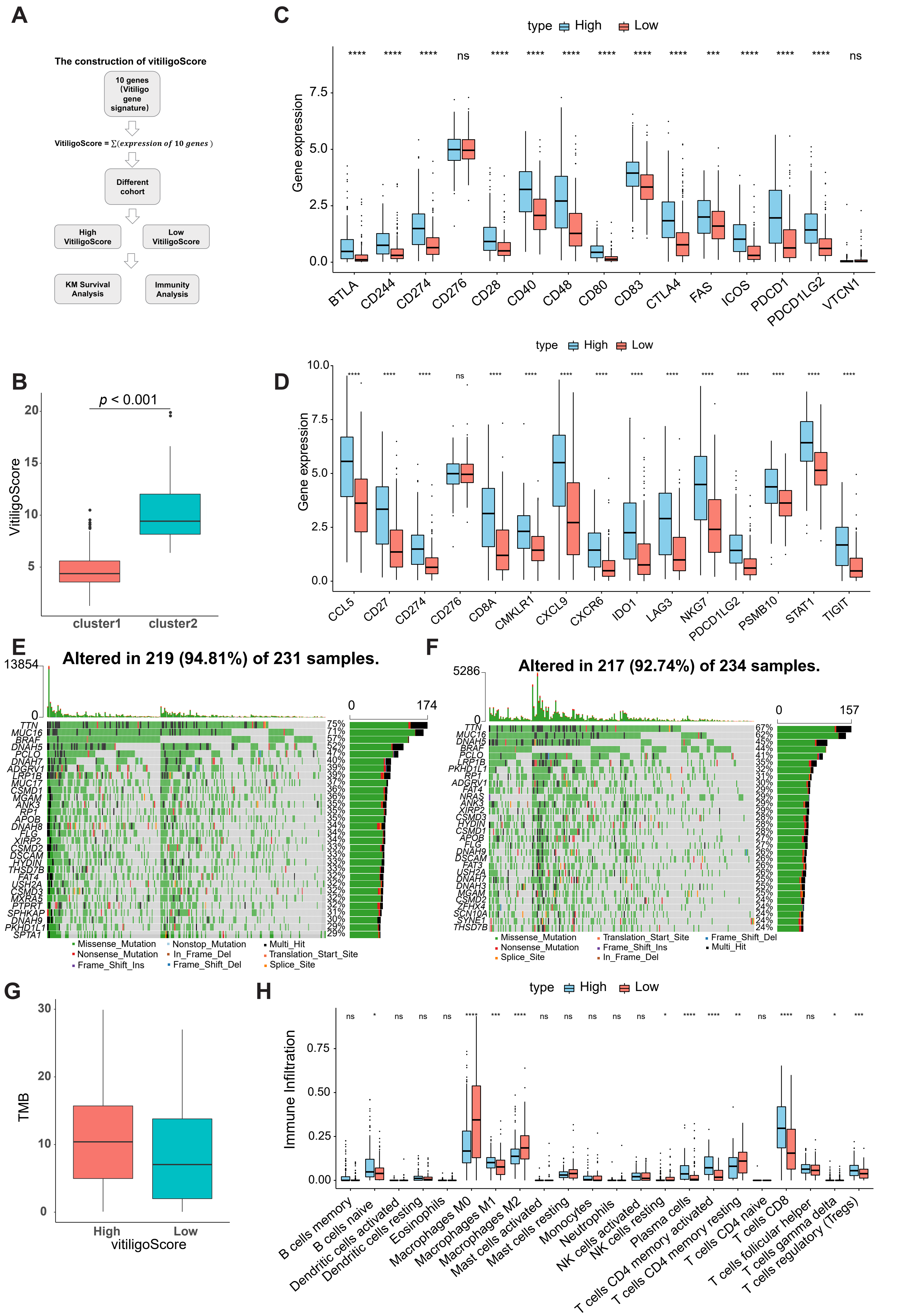

Figure S3. Vitiligo score was significantly related to immunity. A, the process of constructing the vitiligo score (VS). B, the VS comparison between the cluster1 and cluster2 (Wilcoxon test). C, the expression of check point genes between high VS group and low VS group, and the statistical significance was assessed by Wilcoxon test (*p < 0.05, **p < 0.01, ***p < 0.001 and ****p < 0.0001). D, the expression of genes of T cell-inflamed GEP, and the statistical significance was assessed by Wilcoxon test (*p < 0.05, **p < 0.01, ***p < 0.001 and ****p < 0.0001). E, the mutations in the high VS group. F, the mutations in the low VS group. G, the comparison of TMB score between high and low VS groups. H, the comparison of immune cells between high and low VS group.
