# Supplementary material for: Vitiligo Signature‐Based Drug Screening Identifies Fulvestrant as a Novel Immunotherapy Combination Strategy
Source: Adv Sci (Weinh). 2025 Sep 20;12(44):e03979. doi: 10.1002/advs.202503979 (PMC12667482; doi:10.1002/advs.202503979)
Supplement: Supplementary file 2 — Supplemental Figures [file ADVS-12-e03979-s001.zip › advs71623-sup-0005-FigureS4.pdf]

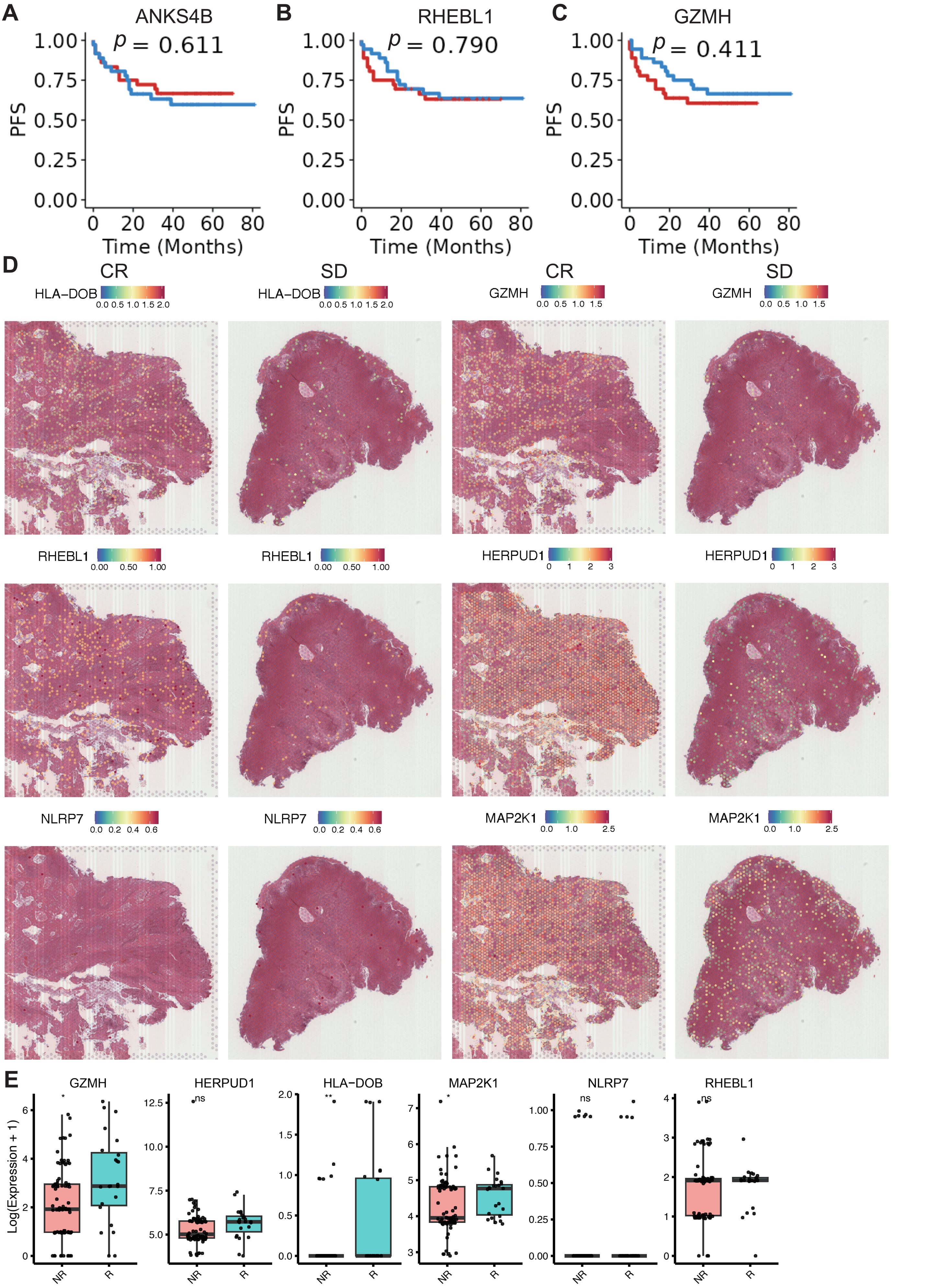

Figure S4. Responder patients in immunotherapy exhibited higher expression of VGS compared with non-responder patients. A-C, the progression-free survival of melanoma patients stratified by the expression of ANKS4B (A), RHEBL1 (B) and GZMH (C). Log-rank test. D, the gene expression of HLA-DOB, GZMH, RHEBL1, HERPUD1, NLRP7, and MAP2K1 in complete response (CR) and stable disease (SD) patients. Boxplots show the expression of six VGS genes in immunotherapy responders (R) versus non-responders (NR) in the Hugo et al. dataset. Expression values are shown as Log(Expression + 1). Statistical comparisons were performed using the Wilcoxon rank-sum test ($p < 0.05$; $p < 0.01$; $p < 0.001$; ns = not significant).
