# Supplementary material for: Vitiligo Signature‐Based Drug Screening Identifies Fulvestrant as a Novel Immunotherapy Combination Strategy
Source: Adv Sci (Weinh). 2025 Sep 20;12(44):e03979. doi: 10.1002/advs.202503979 (PMC12667482; doi:10.1002/advs.202503979)
Supplement: Supplementary file 2 — Supplemental Figures [file ADVS-12-e03979-s001.zip › advs71623-sup-0006-FigureS5.pdf]

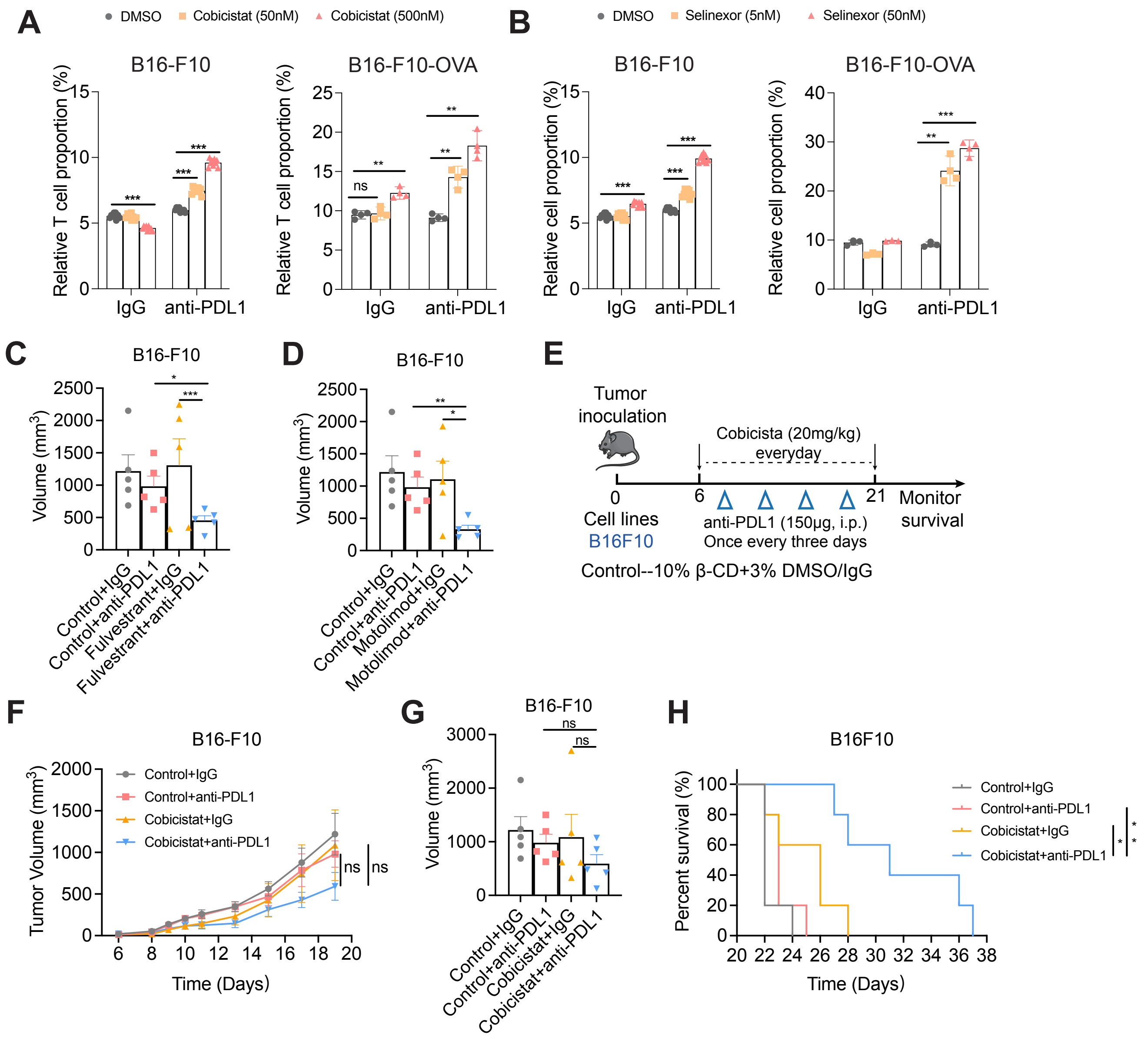

Figure S5. The impact of the combination of Cobicistat and Selinexor with PD-L1 on tumor progression. A, Relative T cell proportion in B16-F10 cell and B16-F10-OVA cell treated with 50nM and 500nM Cobicistat for 48h. B, Relative T cell proportion in B16-F10 cell and B16-F10-OVA cell treated with 5nM and 50nM Selinexor for 48h. C-D, Tumor sizes at day 19 (sample-paired Student's t-test). *p < 0.05, **p< 0.01, ***p < 0.001, and ****p < 0.0001. Error bars depict SEM. E, Illustration of animal models. B16-F10 cells were injected into C57BL mice. Animals were administrated when the volumes of tumors were about 50 mm³. F, The growth of B16-F10 tumors was measured by tumor volume; volume (mm^3) = [width^2 (mm^2) × length (mm)]/2. G, Tumor sizes at day 19 (sample-paired Student's t-test). *p< 0.05, **p < 0.01, ***p < 0.001, and ****p < 0.0001. Error bars depict SEM. H, Kaplan-Meier plots demonstrating the association between B16-F10 tumors and overall survival. Log-rank test.
