# Supplementary material for: Vitiligo Signature‐Based Drug Screening Identifies Fulvestrant as a Novel Immunotherapy Combination Strategy
Source: Adv Sci (Weinh). 2025 Sep 20;12(44):e03979. doi: 10.1002/advs.202503979 (PMC12667482; doi:10.1002/advs.202503979)
Supplement: Supplementary file 2 — Supplemental Figures [file ADVS-12-e03979-s001.zip › advs71623-sup-0007-FIgureS6.pdf]

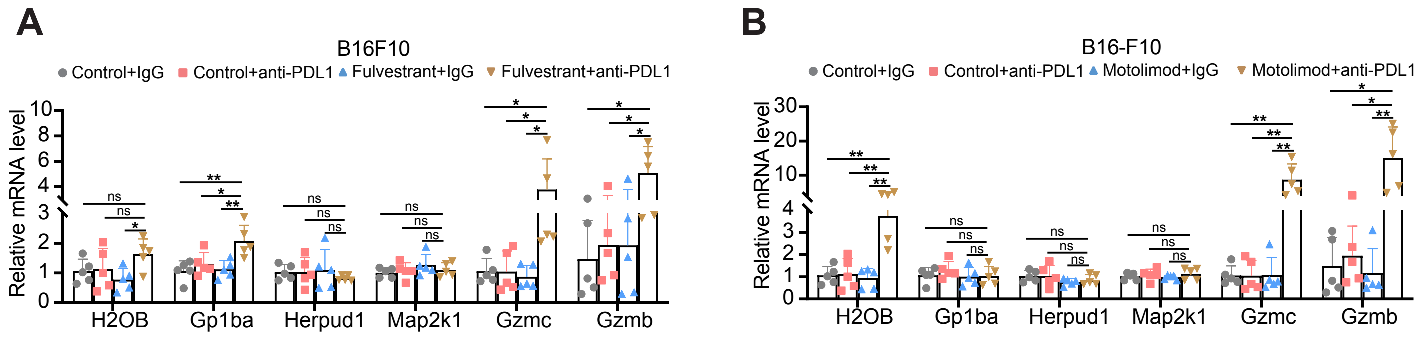

Figure S6. Fulvestrant modulates VGS gene expression in B16-F10 cells. A–B, Gene expression analysis showing the transcriptional changes of vitiligo gene signature (VGS) components in B16-F10 tumor cells following treatment with (A) Fulvestrant and (B) Motolimod. Data demonstrate the impact of each drug on VGS-related genes involved in immune regulation and potential response to immunotherapy.
