# Supplementary material for: Vitiligo Signature‐Based Drug Screening Identifies Fulvestrant as a Novel Immunotherapy Combination Strategy
Source: Adv Sci (Weinh). 2025 Sep 20;12(44):e03979. doi: 10.1002/advs.202503979 (PMC12667482; doi:10.1002/advs.202503979)
Supplement: Supplementary file 2 — Supplemental Figures [file ADVS-12-e03979-s001.zip › advs71623-sup-0008-FigureS7.pdf]

A

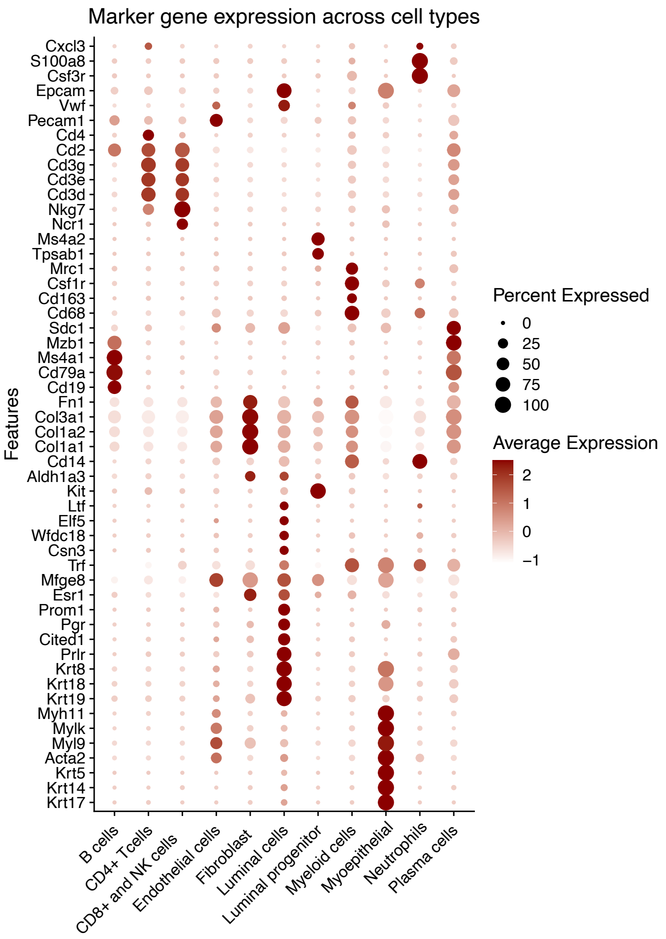

Figure S7. Dot plot showing canonical marker gene expression used to define major cell types. The dot plot visualizes the expression patterns of representative marker genes across different cell clusters. Dot size reflects the percentage of cells expressing the gene within each cluster, and color intensity indicates the average expression level.
