# Supplementary material for: Vitiligo Signature‐Based Drug Screening Identifies Fulvestrant as a Novel Immunotherapy Combination Strategy
Source: Adv Sci (Weinh). 2025 Sep 20;12(44):e03979. doi: 10.1002/advs.202503979 (PMC12667482; doi:10.1002/advs.202503979)
Supplement: Supplementary file 2 — Supplemental Figures [file ADVS-12-e03979-s001.zip › advs71623-sup-0010-FigureS9.pdf]

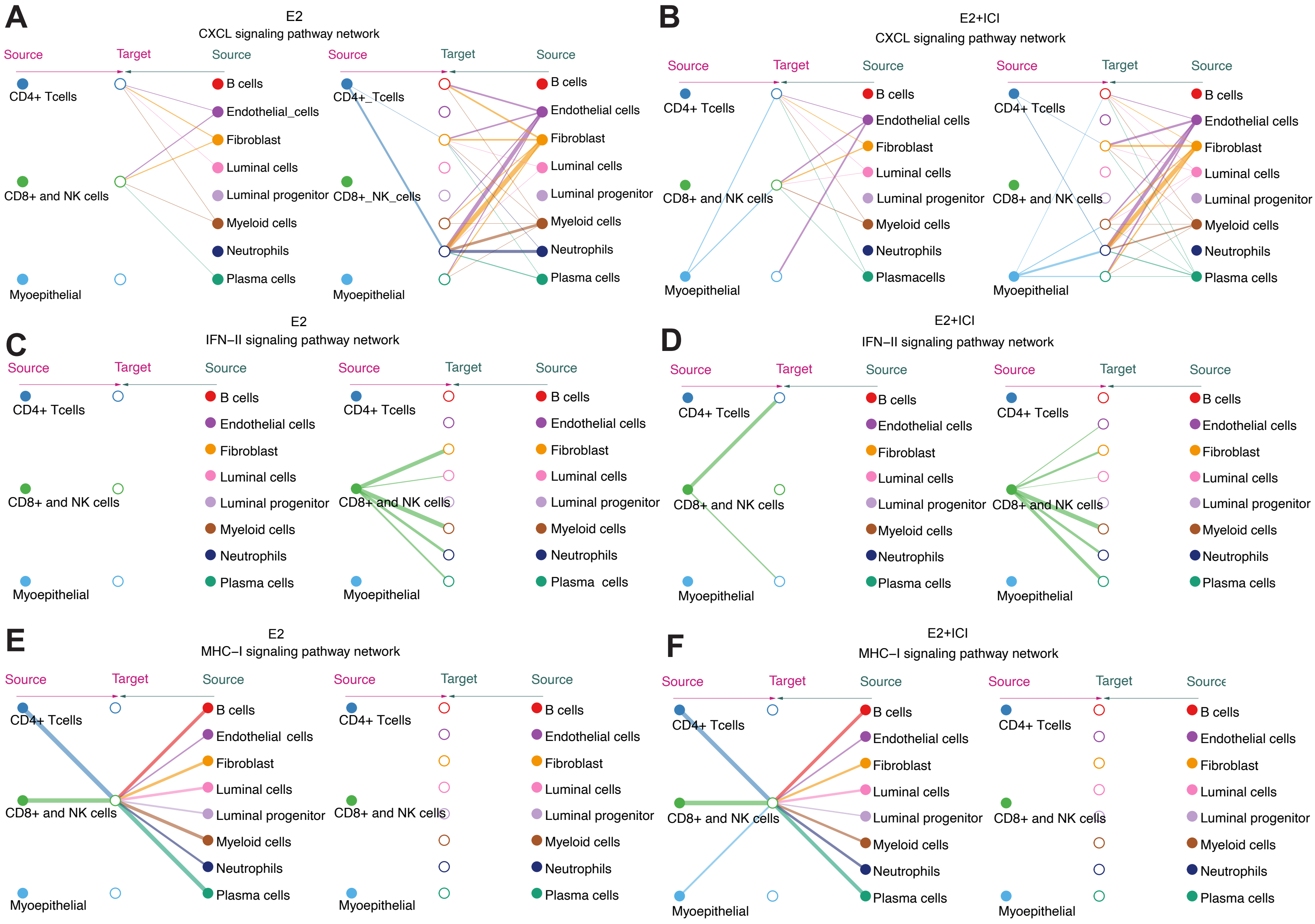

Figure S9. Fulvestrant significantly activated the antigen processing and presentation signaling pathways. A-C, the differences of cell communication strength between E2 and E2+ICI group in CXCL, IFN-II, and MHC-I signaling pathways. After Fulvestrant perturbation, the cell communication of the above three pathways was increased for the cytotoxic T cells (CD8+ and NK cells) and myoepithelial cells.
