# Supplementary material for: Vitiligo Signature‐Based Drug Screening Identifies Fulvestrant as a Novel Immunotherapy Combination Strategy
Source: Adv Sci (Weinh). 2025 Sep 20;12(44):e03979. doi: 10.1002/advs.202503979 (PMC12667482; doi:10.1002/advs.202503979)
Supplement: Supplementary file 2 — Supplemental Figures [file ADVS-12-e03979-s001.zip › advs71623-sup-0011-FigureS10.pdf]

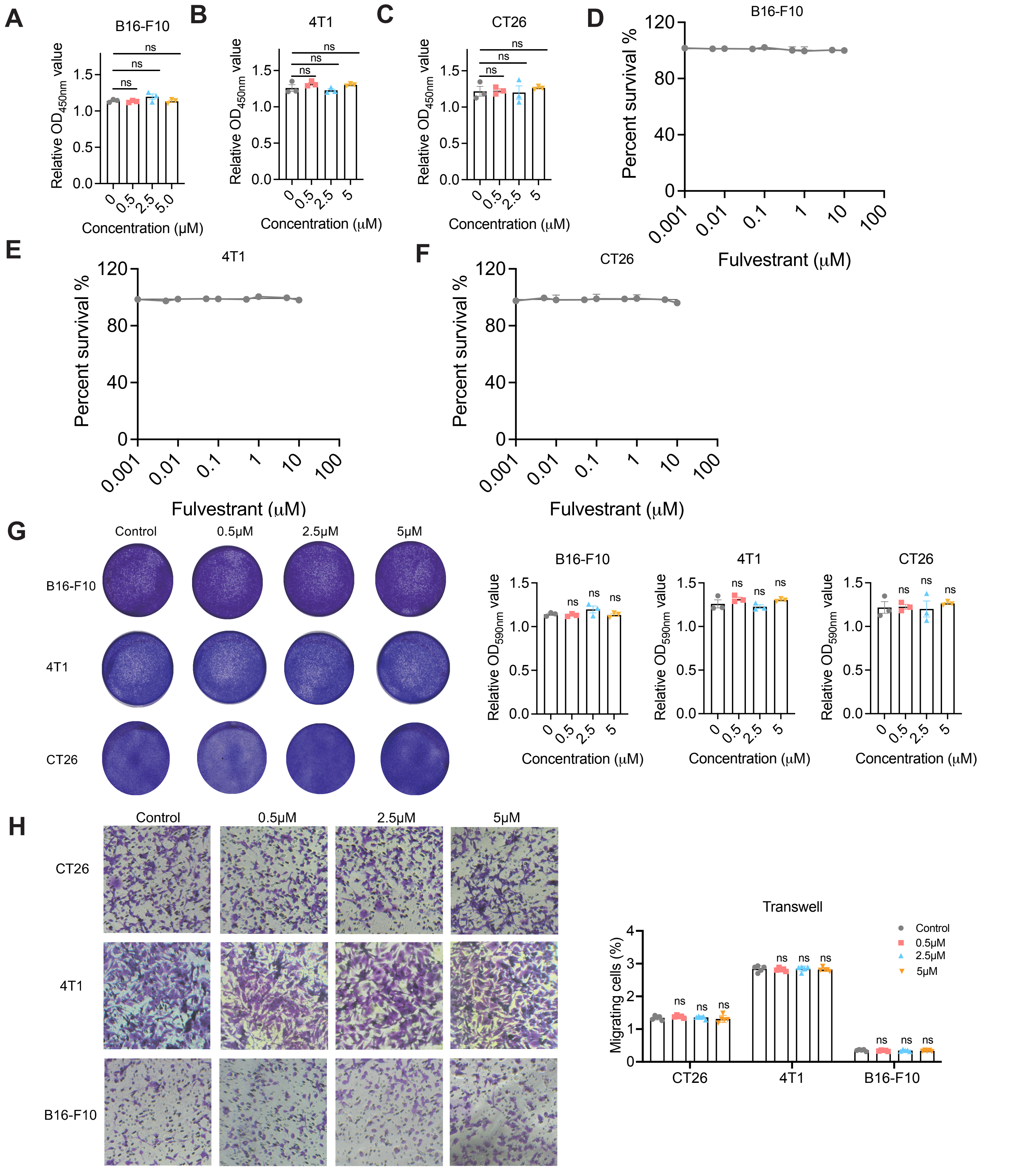

Figure S10. Fulvestrant does not directly impair tumor cell viability, proliferation, or migration in vitro. A–C, bar plots showing the viability of B16-F10, 4T1, and CT26 tumor cell lines after treatment with different doses of Fulvestrant, assessed by CCK-8 assay. D-F, Dose–response curves showing the viability of B16-F10, 4T1, and CT26 tumor cell lines after treatment with Fulvestrant, assessed by CCK-8 assay. G, Crystal violet staining and quantification of colony formation after treatment with different concentrations of Fulvestrant in B16-F10, 4T1, and CT26 cells. No significant differences were observed. H, Transwell migration assay showing that Fulvestrant treatment (0.5, 2.5, and 5μM) does not significantly affect the migration capacity of B16-F10, 4T1, or CT26 tumor cells. Data are shown as mean ± SEM from at least three independent experiments. ns = not significant.
