# Supplementary material for: Vitiligo Signature‐Based Drug Screening Identifies Fulvestrant as a Novel Immunotherapy Combination Strategy
Source: Adv Sci (Weinh). 2025 Sep 20;12(44):e03979. doi: 10.1002/advs.202503979 (PMC12667482; doi:10.1002/advs.202503979)
Supplement: Supplementary file 2 — Supplemental Figures [file advs71623-sup-0009-FigureS8.pdf › advs71623-sup-0009-FigureS8.pdf]

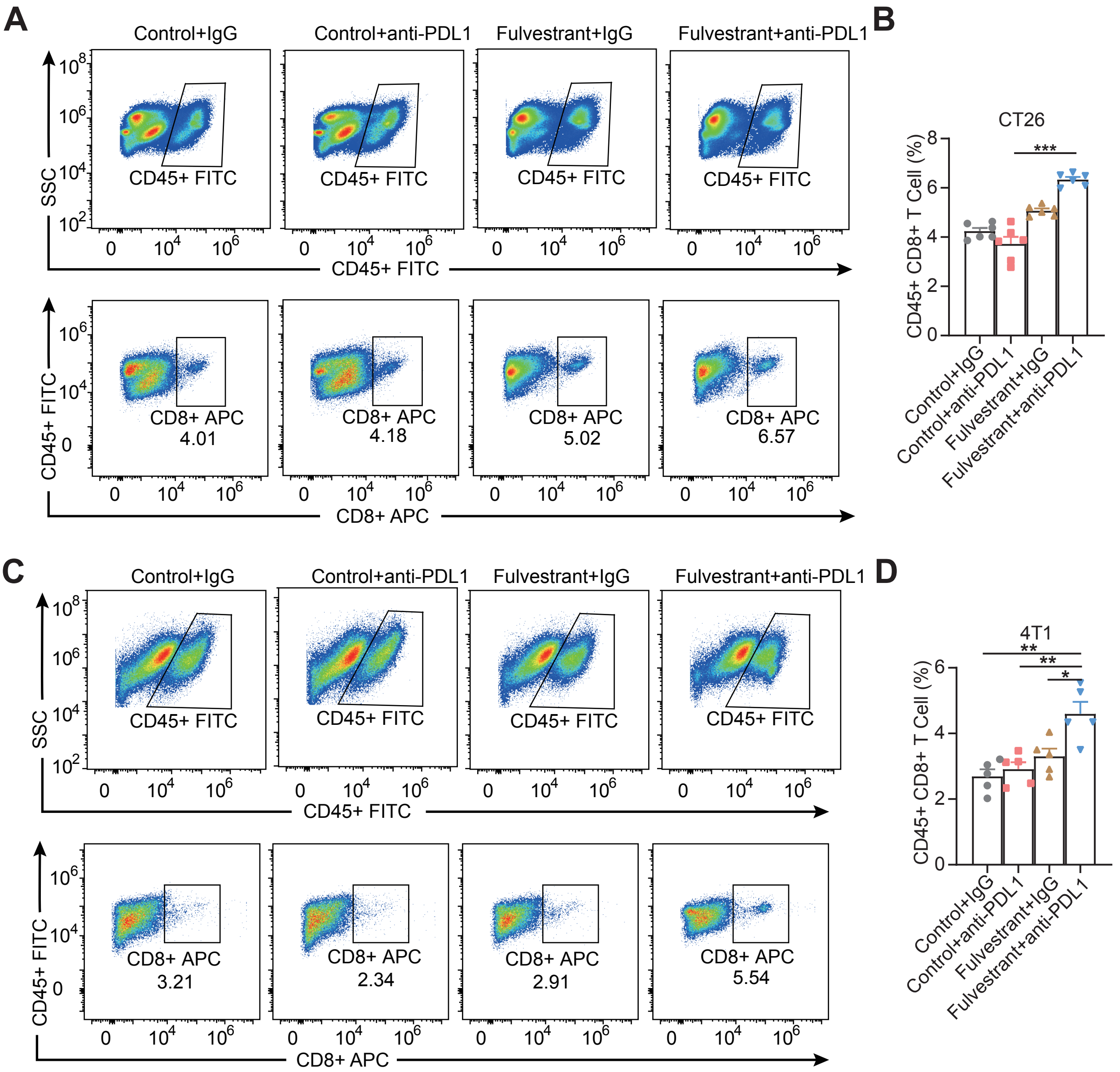

Figure S8. The effects of Fulvestrant immunotherapy on immune cells. (A-B) Representative flow cytometry plots of CD8+ cells (A) and the percentages of CD8+ cells within the CD45+ cell population (B) in CT26 xenograft tumors (n=6) after four injections of PD-L1 antibody in different treatment groups. (C-D) Representative flow cytometry plots of CD8+ cells (C) and the percentages of CD8+ cells within the CD45+ cell population (D) in 4T1 xenograft tumors (n=5) after four injections of PD-L1 antibody in different treatment groups. One-way ANOVA was used to determine statistical significance. Data are presented as mean ± SEM. *p<0.05; **p<0.01; ***p<0.001; ****p<0.0001.
